# Supplementary material for: High temperature Néel skyrmions in simple ferromagnets
Source: Nat Commun. 2026 Jun 2;17:4911. doi: 10.1038/s41467-026-73775-w (PMC13234276; doi:10.1038/s41467-026-73775-w)
Supplement: Supplementary file 1 — Supplementary Information [file 41467_2026_73775_MOESM1_ESM.pdf]

Supplementary Information

High temperature Néel skyrmions in simple ferromagnets

Peng Wang[1], Rana Saha[1,2], Holger L. Meyerheim[1], Ke Gu[1], Hakan Deniz[1], David Eilmsteiner[3], Andrea Migliorini[1], Banabir Pal[1,4], Juan Rubio Zuazo[5,6], Engenia Sebastiani-Tofano[5,6], Ilya Kostanovski[1], Abhay Kant Srivastava[1], Arthur Ernst[1,3], Stuart S. P. Parkin[1,4*]

[1]Max Planck Institute of Microstructure Physics, Weinberg 2, 06120 Halle (Saale), Germany

[2]Department of Chemistry, Indian Institute of Science Education and Research, Yerpedu, Tirupati 517619, India

[3]Institute for Theoretical Physics, Johannes Kepler University Linz, Altenberger Strasse 69, A-4040, Linz, Austria

[4]Halle-Berlin-Regensburg Cluster of Excellence CCE, Halle (Saale), Saxony-Anhalt, Germany

[5]Spanish CRG Beamline BM25-SpLine at the ESRF, 38043 Grenoble, France

[6]Instituto de Ciencia de Materiales de Madrid-CSIC, 28049, Madrid, Spain

*e-mail: stuart.parkin@mpi-halle.mpg.de

Growth of Co_xAl and Co_xNi_yAl thin films

A series of Co_xAl alloy thin films were deposited using magnetron co-sputtering from cobalt (Co) and aluminum (Al) targets. To obtain $Co_{2.3}Al$ films of varying thicknesses, the sputtering duration was systematically adjusted.

All the bilayer thin film samples were grown on 10×10 mm^2 MgO (001) substrates with a 2 nm thick MgO as a buffer layer, and the films are capped with a 5 nm thick highly resistive TaN (ρ~1000 μΩ cm) as a protective layer to prevent the thin films from oxidation. And the single layer t Co_xAl films used for composition analysis by Rutherford backscattering spectroscopy (RBS) and sheet resistance measurement by Veeco FPP 5000 four-point probe were also grown on 10×10 mm^2 MgO (001) substrates with a 2 nm thick MgO as a buffer layer but have a 4 nm thick MgO as a capping layer. The growth process for the bilayer 4.3 nm IrAl | 30 nm Co_xAl heterostructures is detailed in the Methods section. The cobalt content was controlled by varying the sputtering power applied to the Co target, while maintaining a constant sputtering power of 95 W for the Al target.

Similarly, 4.3 nm IrAl | 27 nm $Co_{2.58}Ni_{0.26}Al$ bilayer thin film structures were grown using the same substrate, buffer and TaN capping layers. The Co_xNi_yAl composition was obtained by adjusting the sputtering power of the Co target, while keeping the Al and Ni target powers fixed at 95 W and 47 W, respectively.

Compositional analysis using Rutherford backscattering spectroscopy (RBS)

RBS analysis was used to determine the composition of the deposited film samples using an Ion Beam Analysis system from National Electrostatic Corporation. Measurements were conducted with a helium ion (He+) beam at an energy of 1.9 MV, employing the Cornell geometry with a scattering angle of 169°. To minimize channeling effects, the sample was positioned with a slight off-axis rotation of 9°. Each RBS spectrum was acquired with a total dose of 40 μQ and a beam current of 20 nA. A typical RBS spectrum is shown in Figure S1**a**, captured using a standard silicon-implanted detector from Ametek. The spectrum reveals elemental peaks of aluminum (Al) and cobalt (Co), along with magnesium (Mg) and oxygen (O) arising from the substrate. Analysis using the SimNRA 7.1 software yields a composition of Co (2.3 ± 0.05) and Al (1). The film

thickness was estimated to be 305 Å, based on the weighted atomic densities of bulk Co and Al metals.

To further investigate the film's chemical composition and assess potential compositional gradients, we performed high-resolution (HR) RBS depth profiling. This process entailed adjusting the beam energy to 577 keV and employing the IBM geometry with an incidence angle of 25° and a scattering angle of 90°. We acquired the spectrum using a magnetic spectrometer coupled with a microchannel plate (MCP) detector, setting the magnet current to 33 A. This specific magnet current facilitated the simultaneous measurement of both Co and Al peaks within a single run. Despite a slight overlap of the Mg peak from the MgO capping layer with the Al peak, the Co profile remained distinctly visible. Figure S1**b** shows the final spectrum and the fitting results analyzed using the SimNRA 7.1 software. This analysis verified a uniform composition across the film, achieving a depth resolution finer than 1 nm.

Additionally, RBS was used to analyze the composition of Co_xAl films as a function of the sputtering power applied to the Co target (see Figure S1**c**).

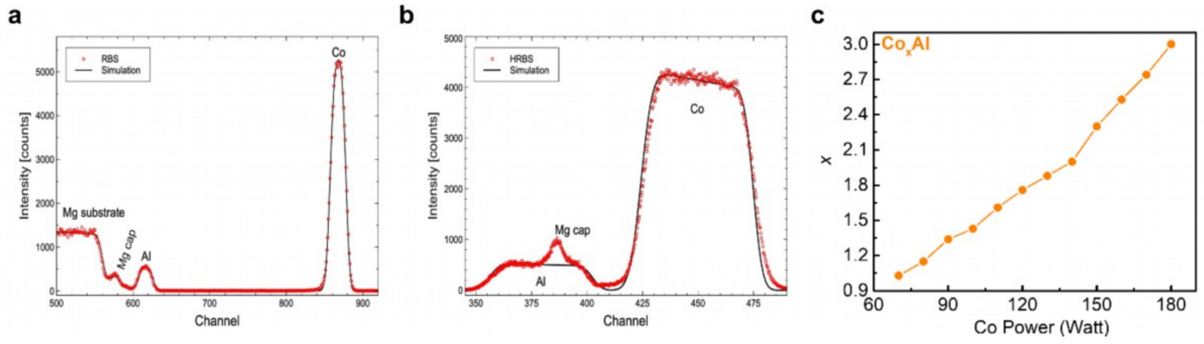

Figure S1: Composition and thickness of Co_xAl thin films. a, RBS Spectrum using a Si-implanted detector and a 1.9 MV He+ beam at a 169° scattering angle, together with a SimNRA simulation for a $Co_{2.3}Al_1$ layer on MgO, capped by MgO. **b**, HR-RBS (high resolution RBS) spectrum obtained using a 577 kV Helium ion beam at a 90° scattering angle, measured with a magnetic spectrometer. A SimNRA simulation of a Co-Al layer on MgO, capped by MgO is shown as a solid line through the experimental data points. **c**, Atomic Ratio, x, of Co to Al in Co_xAl thin films as a function of sputtering power applied to the Co target.

X-ray diffraction analysis

The average c-lattice parameters of the as-grown thin films were characterized using conventional 2θ-θ specular scans using a Bruker-D8 x-ray diffraction (XRD) system with $\lambda_{Cu\text{-}K\alpha}$ = 1.5406. Å. Figure S2 shows typical XRD patterns for a series of Co_xAl films grown on MgO (001) substrate with 2 nm MgO buffer layer and 4 nm thick MgO as capping layer for various x. Figure S2**b** shows how the Co_xAl (002) peak position changes as a function of x. The out-of-plane lattice parameter (c) is derived from the (002) XRD peak position (θ) according to Bragg's equation λ=2$d$sin(θ), where λ is the wavelength (1.5406 Å) and d is the spacing of the diffracting lattice planes. The dependence of c on x is shown in Fig. S2**d**.

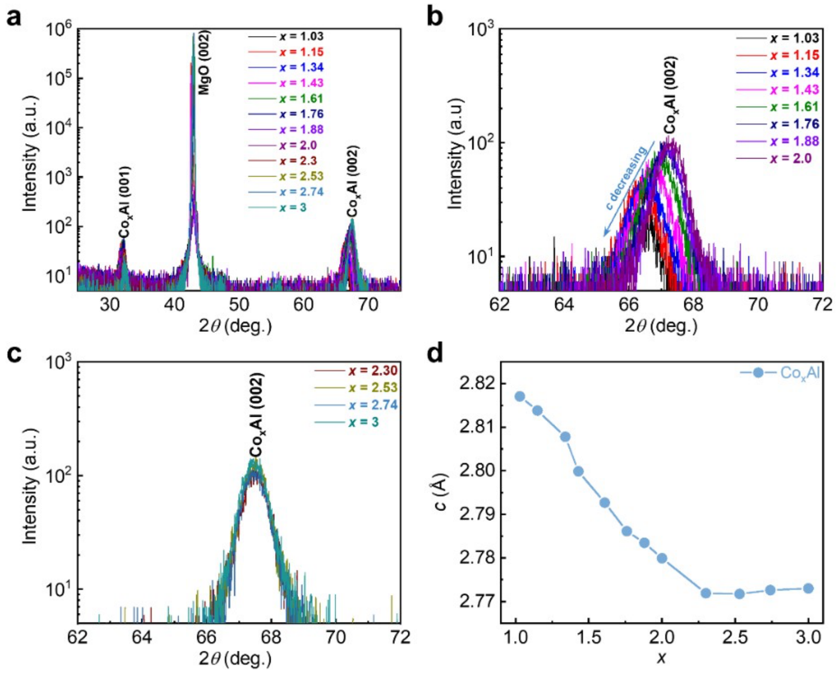

Figure S2: X-ray diffraction measurements of Co_xAl alloy thin films. a-c, XRD 2θ-θ specular scan measurements of Co_xAl films co-sputtered from cobalt and aluminum targets (sputtering time of 300 sec). **d**, Out-of-plane lattice parameter c as a function of x.

The variation of the crystal structure as a function of thickness in single layer $Co_{2.3}Al$ thin films (with 2 nm MgO underlayer without any IrAl buffer layer) capped with 4 nm thick MgO as

protective layer was examined by similar XRD measurements. The XRD 2θ-θ specular scans for different thicknesses are shown in Figure S3**a** and S3**b**.

Similar XRD scans for 4.3 nm IrAl | 27 nm $Co_{2.58}Ni_{0.26}Al$ bilayer thin film are shown in Figure S6.

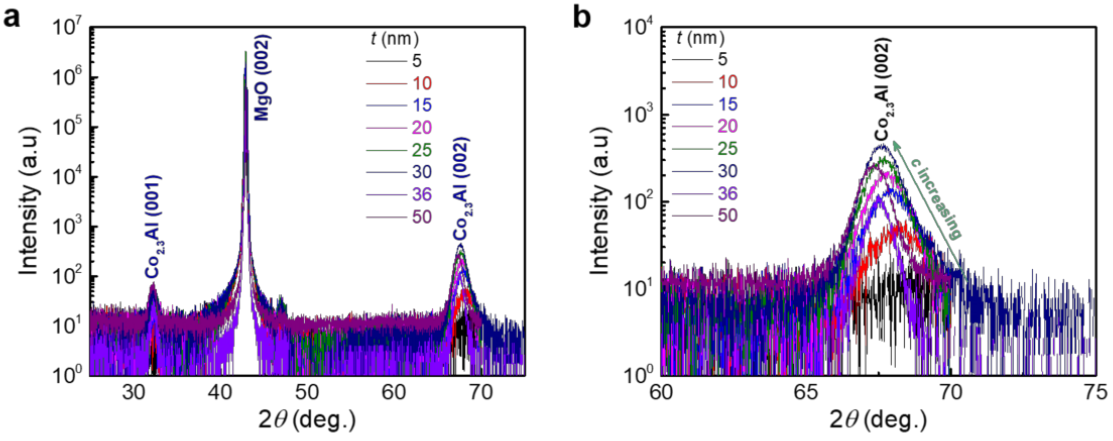

Figure S3: X-ray diffraction measurements of $Co_{2.3}Al$ alloy films with different thicknesses. a, **b**, 2θ-θ specular scans of $Co_{2.3}Al$ thin films with different thicknesses t.

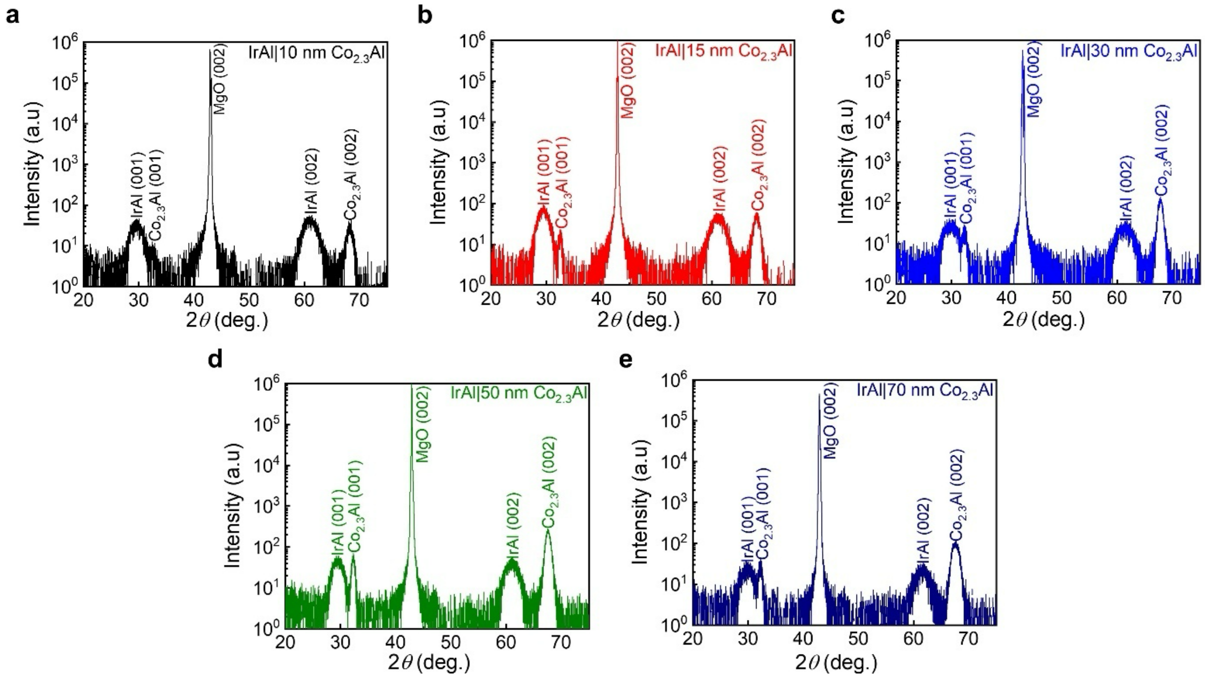

Figure S4: X-ray diffraction analysis of 4.3 nm IrAl | *t* $Co_{2.3}Al$ bilayer thin films versus thickness of the $Co_{2.3}Al$ layer, *t*. a-e, X-ray diffraction measurements (2θ-θ specular scans) as a function of *t* (see SI for details). *t* is given in each figure (top right).

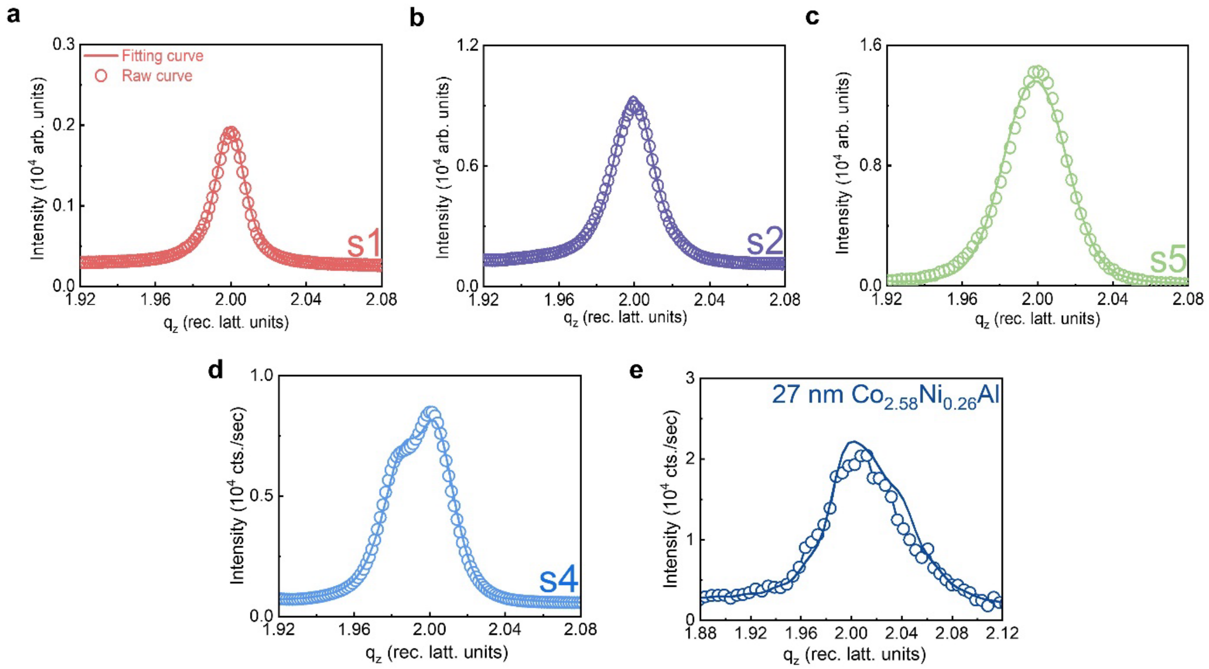

Figure S5: X-ray diffraction peak profiles at ambient temperature. a-e, Experimental (circles) and calculated (solid line) X-ray diffraction profiles on a linear scale in the vicinity of the L=2 reflection along [00L] for samples s1 and s2 and [20L] for sample s5 in reciprocal space. XRD results for sample s3 is given in Fig. 1c of the main manuscript. While the profiles of samples s1 (30 nm $Co_{2.3}Al$) and s5 (4.3 nm IrAl | 70 nm $Co_{2.3}Al$) are nearly symmetric corresponding to a small strain gradient, those of s2 (4.3 nm IrAl | 20 nm $Co_{2.3}Al$), s3 (4.3 nm IrAl | 30 nm $Co_{2.3}Al$), s4 (4.3 nm IrAl | 50 nm $Co_{2.3}Al$) and 4.3 nm IrAl | 27 nm $Co_{2.58}Ni_{0.26}Al$ films exhibit a pronounced asymmetry indicating a significant strain gradient.

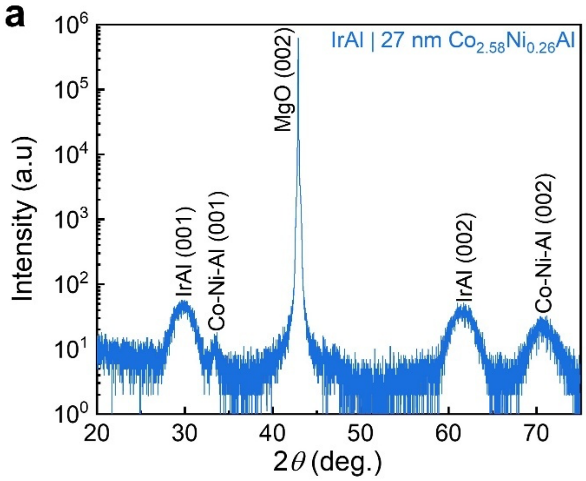

Figure S6: X-ray diffraction measurements of IrAl | 27 nm $Co_{2.58}Ni_{0.26}Al$ bilayer thin film. a, 2θ-θ scans of IrAl | 27 nm $Co_{2.58}Ni_{0.26}Al$ bilayer thin film. The atomic composition and thickness of the IrAl under layer are $Ir_{42}Al_{58}$ and 4.3 nm, respectively.

Vibrating sample magnetometer (VSM) measurements

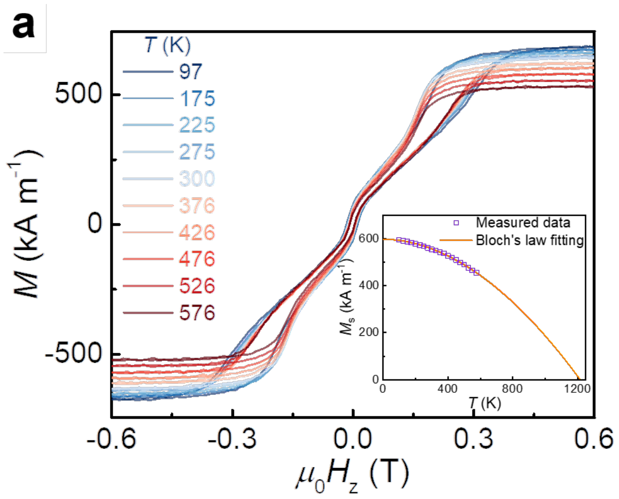

Figure S7: Temperature dependent magnetization measurements. Magnetization (M) versus out of plane magnetic field ($\mu_0 H_z$) for a 4.3 nm IrAl | 50 nm $Co_{2.3}Al$ bilayer at various temperatures between 97 and 576 K. Inset shows saturation magnetization (M_s) as a function of temperature.

We estimated the ferromagnetic transition temperature of an 4.3 nm IrAl | 50 nm $Co_{2.3}Al$ bilayer by measuring out-of-plane magnetic hysteresis loops at different temperatures, in the range of 100

K to 600 K, as shown in Figure S7. With increasing temperature, the saturation magnetization (M_S) decreases, while the hysteresis loop profile is retained up to 600 K. The sample surface area is 0.329 × 0.316 cm^2. Each M_S corresponds to an average over ten measurements. The temperature dependence is fitted to Bloch's law according to:

$$M_s(T) = M_s(0) \times ((1 - (T/T_c)^{\alpha}) \qquad (1)$$

with parameters $M_s(0)$ = 598 kA m^{-1}, T_c = 1216 K and α = 1.92. The value of the exponent α can be accounted for by likely structural defects in the film[1,2].

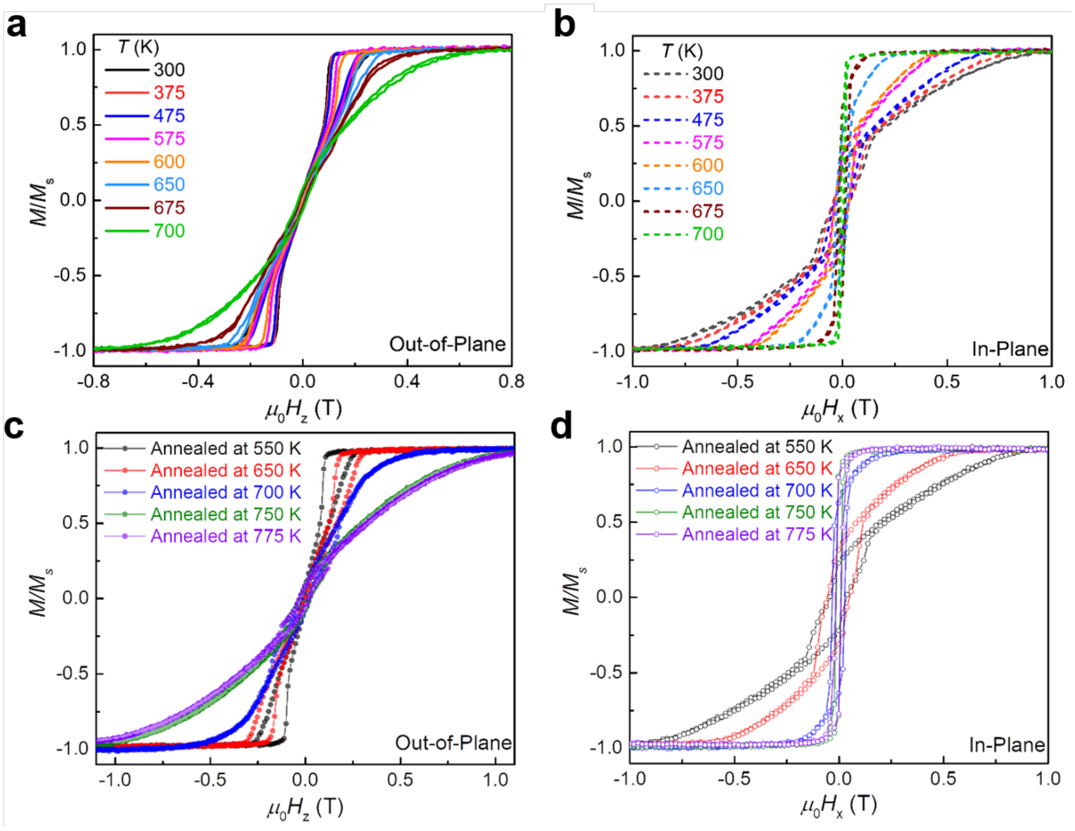

Figure S8: Magnetic characterization of 4.3 nm IrAl | 30 nm $Co_{2.3}Al$ bilayer films. Out-of-plane **a**, and in-plane **b**, normalized magnetization hysteresis loops measured in the temperature range from 300 to 700 K. Out-of-plane **c**, and in-plane **d**, normalized magnetization hysteresis loops measured after sample annealing in Ar at various temperatures in the range from 550 to 775 K.

Temperature dependent *M-H* loops were measured with a Lakeshore 8600 VSM system using the SSVT option under a continuous flow of Ar. The measured curves were background subtracted. The resulting data is flattened to remove the linear response of both the substrate and holder. Firstly, the VSM system was outgassed for about 1 hour at 700 K before inserting the sample. Subsequently, temperature dependent out-of-plane and in-plane *M-H* measurements of a 4.3 nm IrAl | 30 nm $Co_{2.3}Al$ bilayer were carried out (Figures S8**a** and **b**). The magnetic response of the sample is preserved up to 600 K, above which an in-plane component starts to appear, which becomes dominant at a temperature of about 700 K. To test the robustness of the 4.3 nm IrAl | 30 nm $Co_{2.3}Al$ bilayer against high-temperature treatments, a piece of the sample was annealed in a UniTemp RTP-100 annealing furnace in an Ar environment at different temperatures, and the *M-H* loops compared with those taken at room temperature (Figures S8**c** and **d**). An irreversible and progressive evolution of the magnetic response upon annealing above 650 K takes place, which finally leads to a complete in-plane easy axis magnetization after annealing at 750 K. The discrepancy between the data from the high temperature treatments in the VSM and the furnace is likely due to systematic errors in measurement of the temperature. However, we cannot exclude the possibility that the film may suffer from partial oxidation during the annealing processes at high temperatures: we note that the base vacuum of the RTP-100 system is only $\sim 10^{-2}$ Torr, which is much worse than the TEM chamber.

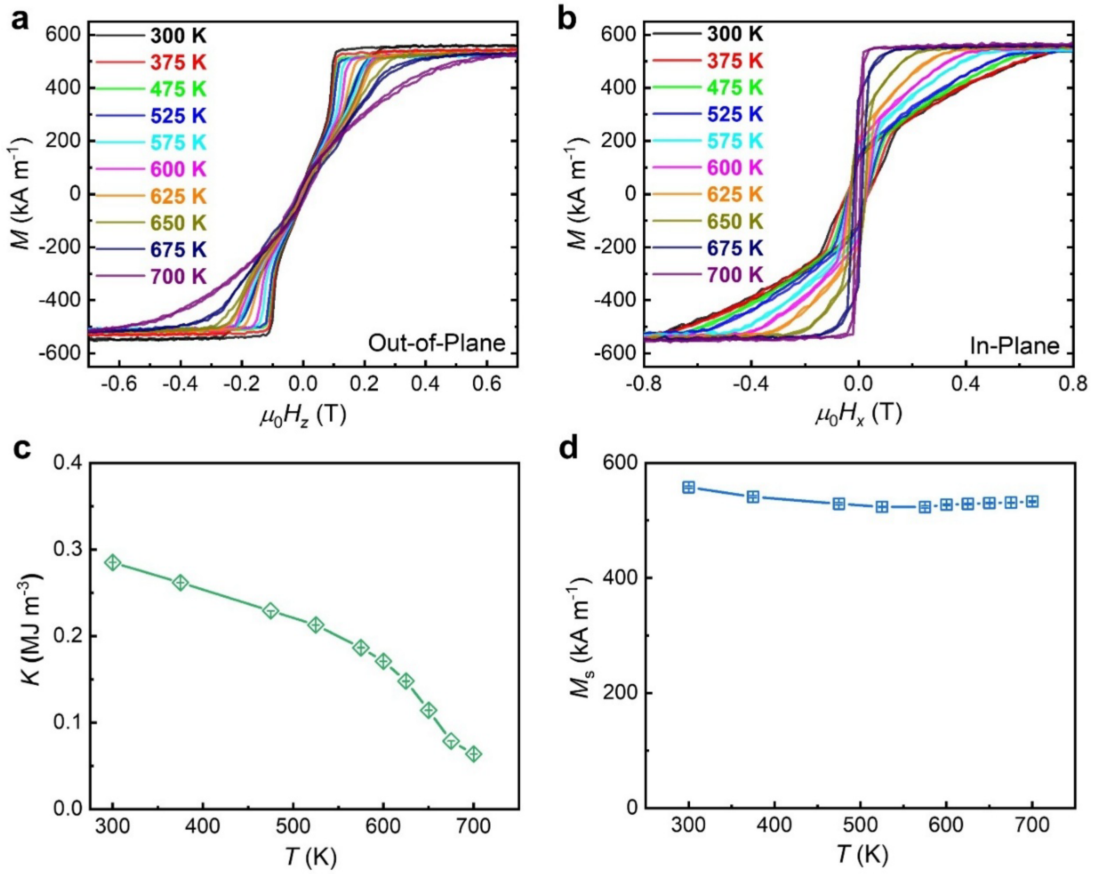

Figure S9: Magnetic characterization of a 4.3 nm IrAl | 30 nm $Co_{2.3}Al$ bilayer thin film versus temperature. Out-of-plane **a** and in-plane **b** magnetization hysteresis loops in the measurement temperature range between 300 and 700 K. Perpendicular magnetic anisotropy (*K*) **c**, and saturation magnetization (M_s) **d**, versus measurement temperature. Error bars are averaged from 5 data points obtained from the saturation magnetization.

Lorentz Transmission Electron Microscope (LTEM)

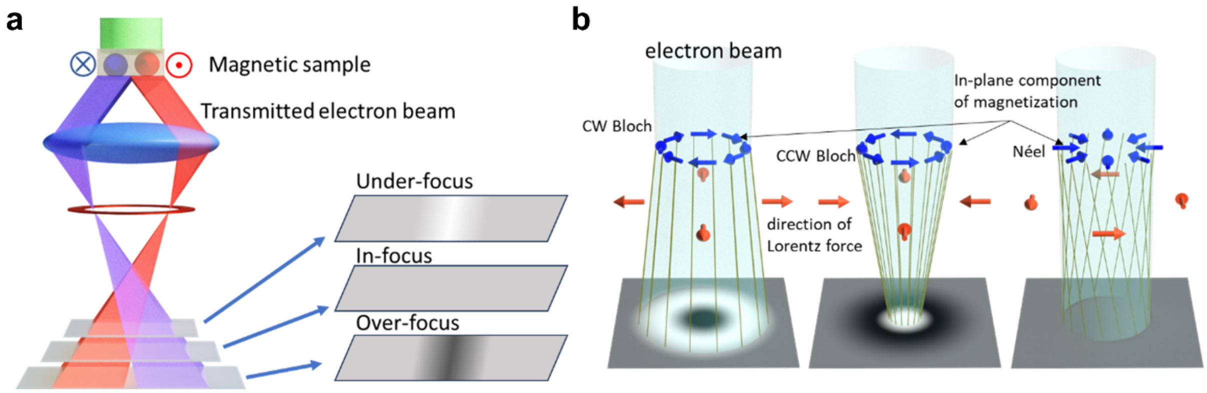

Figure S10: **a**, Schematic of the principle of LTEM showing image formation under different defocus conditions. The magnetic sample shown here contains two opposite domains, whose in-plane magnetic component direction is represented by a blue cross and a red dot. **b**, Schematic illustration of the electron beam deflection resulting in different contrasts for a clockwise (CW) Bloch skyrmion, a counter-clockwise (CCW) Bloch skyrmion, and a Néel skyrmion. The dark yellow lines in **b** show the approximate trajectory of the transmitted electrons in each case. The blue arrows represent the in-plane component of magnetization for each type of skyrmion, whereas the orange arrows represent the corresponding direction of the Lorentz forces experienced by the transmitted electrons.

A similar working principle is used to image more complex textures, for example skyrmionic spin textures that are illustrated in Figure S10**b**. The in-plane magnetization components for spin-textures corresponding to three distinct types of skyrmions are indicated by blue arrows, while the direction of the deflected electron beam due to the Lorentz force is shown by red arrows. For instance, a clockwise Bloch skyrmion yields a central dark spot surrounded by a bright ring, while a counter-clockwise one produces the reverse contrast. The case of Néel skyrmions is, however, more subtle. Because the in-plane magnetization components are radially symmetric (pointing either inwards or outwards from the center), electron deflections do not create any visible contrast in the LTEM image when the sample is at normal incidence to the electron beam (zero tilt condition).

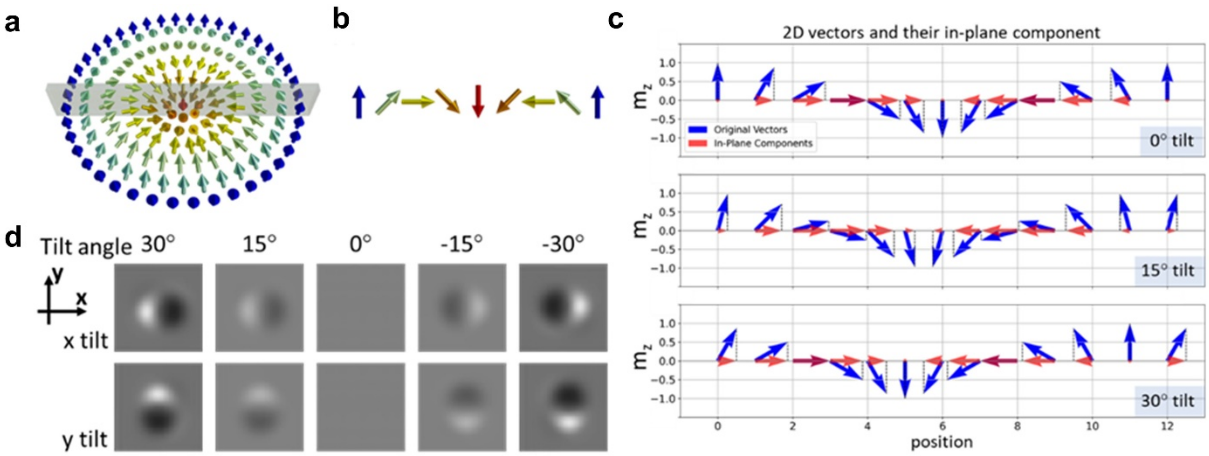

Figure S11: **a**, Schematic diagram showing the spin texture of a Néel skyrmion, where the arrows represent the direction of the magnetic moments. **b**, Cross-section of the Néel skyrmion as shown in a, taken along the shaded area in a. **c**, Representations of the in-plane component of the magnetic moments in the cross-section of a Néel skyrmion under different tilt conditions. Blue arrows in **c** indicate the direction of moments, while the direction and length of the red arrows represent the corresponding direction and magnitude of their in-plane components of magnetization, respectively. **d**, Simulated LTEM contrast of a Néel skyrmion under different tilting conditions.

However, tilting of the sample breaks this symmetry, as shown in Figure S11. A cross-section of a Néel skyrmion reveals that, upon tilting, the in-plane magnetization components become imbalanced across the beam path, producing a net deflection. As a result, contrast appears. We show that the simulated images at positive and negative tilting angles (0°, 15°, 30°, -15°, and -30°), where the contrast increases with increasing tilt angle, as the effective in-plane magnetization component grows (Figure S11**c** and S11**d**). These visualizations are supported by micromagnetic and LTEM image simulations using the open-source PyLorentz (https://github.com/PyLorentz/PyLorentz) software. Simulated LTEM contrast images for an isolated Néel skyrmion under different tilting angles clearly show that the contrast appears only under tilted conditions and increases with the degree of tilting. The resulting contrast is circular and divided into bright and dark halves, consistent with theoretical expectations.

LTEM analysis of selected samples

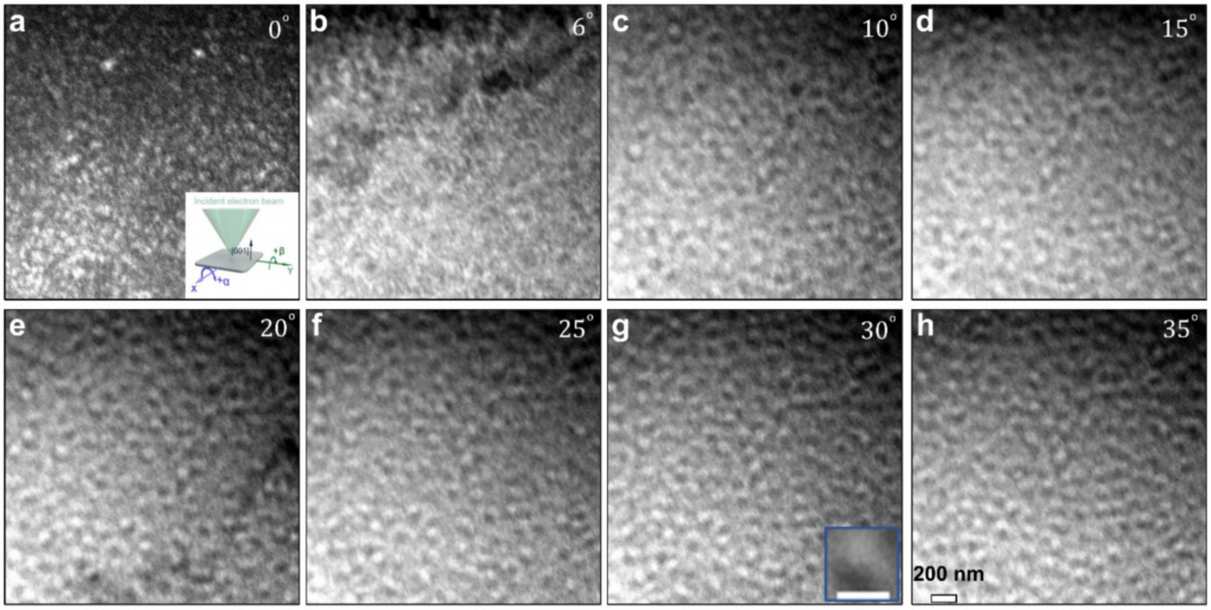

Figure S12: Tilt angle dependent Lorentz transmission electron microscopy (LTEM) images of 4.3 nm IrAl | 30 nm $Co_{2.3}Al$ showing Néel skyrmions. a-h, LTEM images of a bilayer of 4.3 nm IrAl | 30 nm $Co_{2.3}Al$ under different tilt angle about the *x*-axis (α) at 300 K in the presence of an 0.15 T out-of-plane magnetic field. All images are recorded at a defocus distance of 1.5 mm. In all images the scale bar is 200 nm, but the scale bar shown in the inset of **g** is 100 nm.

In Figure S12 we show tilt angle (α) dependent LTEM images of a 4.3 nm IrAl **|** 30 nm $Co_{2.3}Al$ bilayer at room temperature. At α=0° no magnetic contrast is observed (Figure S12**a**). A magnetic contrast consistent with a Néel skyrmion (circular region with half-dark and half-bright regions at opposite edges) is observed at $\alpha \geq 10°$ (Figures S12**c**, **d**). At α=0° the in-plane components of the magnetic moments within the Néel skyrmions are aligned either radially outward or inward and therefore deflect the incoming electrons in such a way that there is no intensity variation in the defocused image plane, thus producing no magnetic contrast. On the other hand, upon tilting the specimen there is a Lorentz force induced asymmetric deflection of the electrons from the projection of magnetization on either side of a Néel skyrmion. As a result, on one side there will be deflected electrons converging hence producing a bright contrast, whilst on the opposite side the diverging deflected electrons will generate a dark contrast. With increasing tilt angle from α=20° to 35° (Figure S12**e-h**), the magnetic contrast gradually increases. The enhanced magnetic contrast with increasing tilt angle is due to the increasing in-plane magnetic

component of the Néel skyrmion resulting in an enhanced deflection of the transmitted electrons owing to the Lorentz force, where the deflection of electrons is directly proportional to the in-plane magnetic induction created by the sample. The tilt angle dependent LTEM experiment provides unambiguous evidence that the observed magnetic textures are Néel-type.

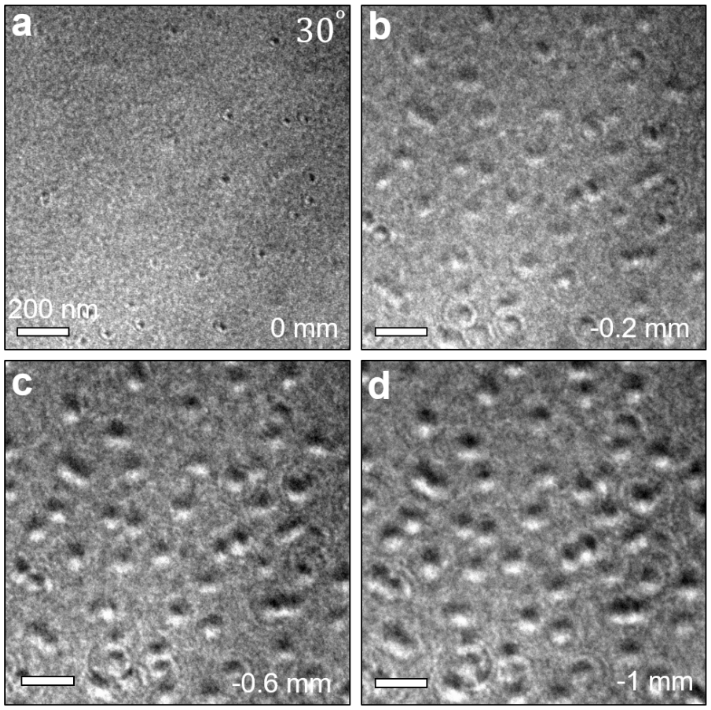

Figure S13: LTEM images of freestanding 4.3nm IrAl | 30nm $Co_{2.3}Al$ at 300 K for different defocus distances. a-d, LTEM images of IrAl | $Co_{2.3}Al$ showing Néel type skyrmions for defocus distances of 0 mm **a**, -0.2 mm **b**, -0.6 mm **c**, and -1.0 mm **d**, in the presence of a 0.25 T magnetic field at a tilt angle of α = 30º (*x*-axis). Scale bar is 200 nm.

Defocus distance dependent LTEM images of a freestanding 4.3 nm IrAl | 30 nm $Co_{2.3}Al$ bilayer sample were recorded under an applied field of 0.25 T applied along the [001] direction, while the specimen is tilted by α=30°. In Figure S13**a**, we show the in-focus image that shows no magnetic contrast. Since Lorentz deflection is extremely small (typically less than 100 μrad), defocusing is required to create a net intensity modulation, enabling the visualization of magnetic contrast in the defocused imaging plane.[3]

By varying the strength of the electromagnetic lens, the viewing plane is moved either up (under-focus) or down (over-focus) with respect to the in-focus plane. The distance between the in-focus and out-of-focus image plane is referred to as the defocus distance, often denoted as $-\Delta f$ (under-focus condition) or $+\Delta f$ (over-focus condition). In Figures S13**b-d**, we show under-focus LTEM images while varying the defocus distance from -0.2 to -1 mm. At -0.2 mm defocus, a weak magnetic contrast characteristic of a Néel skyrmion is observed (Figure S13**b**). With increasing defocus distance, the magnetic contrast becomes much stronger, as observed from the LTEM micrographs (Figures S13**c** and **d**). The nature of the magnetic contrast (circle with half dark and half white at opposite edges) does not change with increasing defocus. The similar LTEM contrast collected in this range of defocus values further confirms that the contrast is characteristic of Néel-skyrmions.

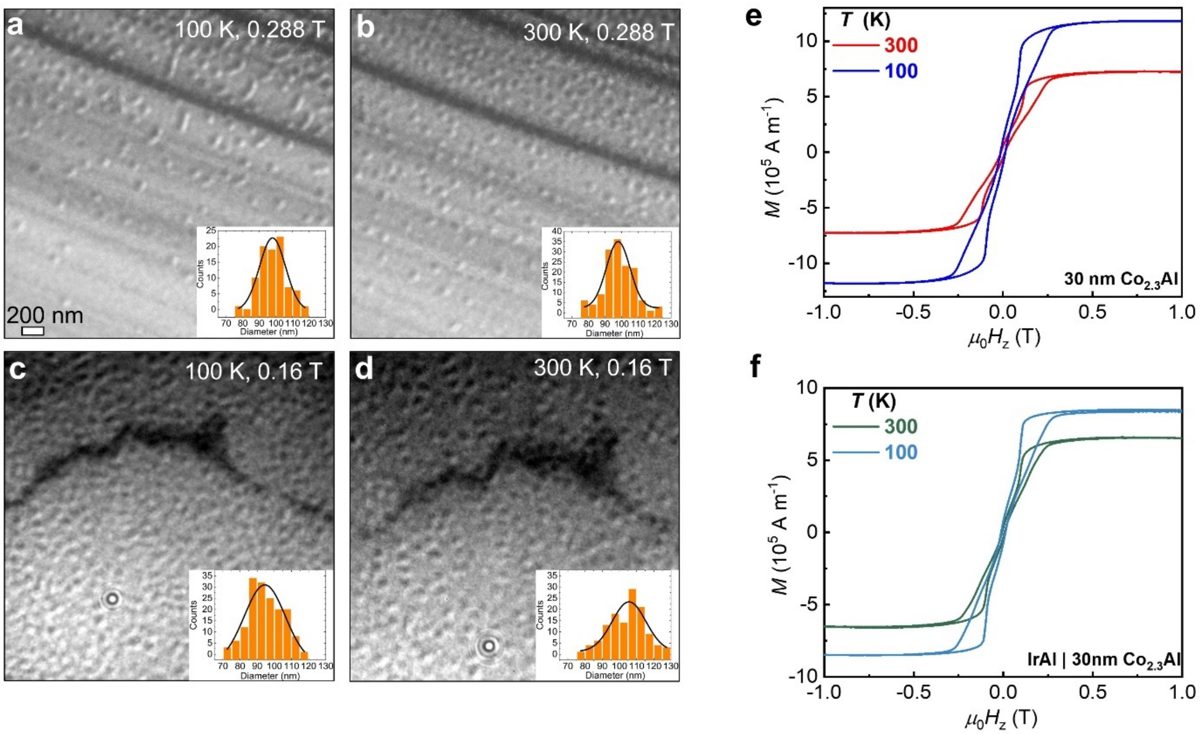

Figure S14: **LTEM images and isothermal magnetization hysteresis plots of 30 nm $Co_{2.3}Al$ single layer showing bubbles and 4.3 nm IrAl | 30 nm $Co_{2.3}Al$ bilayer showing Néel skyrmions at 100 and 300 K.** **a**-**d**, LTEM images of a 30 nm $Co_{2.3}Al$ single layer under an out-of-plane magnetic field of 0.288 T (**a**, and **b**), and of a 4.3 nm IrAl | 30 nm $Co_{2.3}Al$ bilayer under an out-of-plane magnetic field of 0.16 T (**c**, and **d**). **e**,**f**, Isothermal magnetization measurements with an out-of-plane magnetic field applied along [001] at 100

and 300 K for both a 30 nm $Co_{2.3}Al$ single layer and a 4.3 nm IrAl | 30 nm $Co_{2.3}Al$ bilayer sample. The inset scale bar in all the LTEM images corresponds to 200 nm.

In Figure S14, we show LTEM images of a single layer of 30 nm $Co_{2.3}Al$ and a bilayer of 4.3 nm IrAl **|** 30 nm $Co_{2.3}Al$ at 300 and 100 K, respectively. In both samples, LTEM images were recorded after zero-field-cooling (ZFC) from room temperature to 100 K. LTEM images were recorded at an under-focus value of -1.5 mm. For the case of the single layer of 30 nm $Co_{2.3}Al$, magnetic contrast appears at zero tilt angle, while for the case of the bilayer of 4.3 nm IrAl **|** 30 nm $Co_{2.3}Al$, magnetic contrast appears only under tilting. We attribute these findings to the presence of achiral type-II magnetic bubble in the former case, and Néel-type skyrmions in the latter case. LTEM images of both samples at $\alpha = 30°$ are compared in Figure S14**a** and **b**. The size (*d*) of the magnetic objects does not change ($d \sim 98$ nm) with temperature (100 K versus 300 K) as shown in the size distribution plot in the inset of Figure S14**a** and **b**. Although the saturation magnetization of 30 nm $Co_{2.3}Al$ at 100 K is nearly doubled as compared to that at 300 K (Figure S14**d**), the effect on the size of the magnetic nanostructures is negligible. In Figure S14**c** and **d**, we show LTEM images of 4.3 nm IrAl **|** 30 nm $Co_{2.3}Al$ under 30° tilting in the presence of out-of-plane magnetic field at 0.16 T, at 100 K and 300 K. These data show that Néel-skyrmions are formed at both temperatures. In this case the size of Néel-skyrmions is slightly smaller ($d = 95$ nm) at 100 K as compared to 300 K ($d = 106$ nm), as shown in the size distribution plot in the inset of Figure S14**c** and **d**. The reduction of the saturation magnetization at 300 K as compared to 100 K is shown in Figure S14**f**.

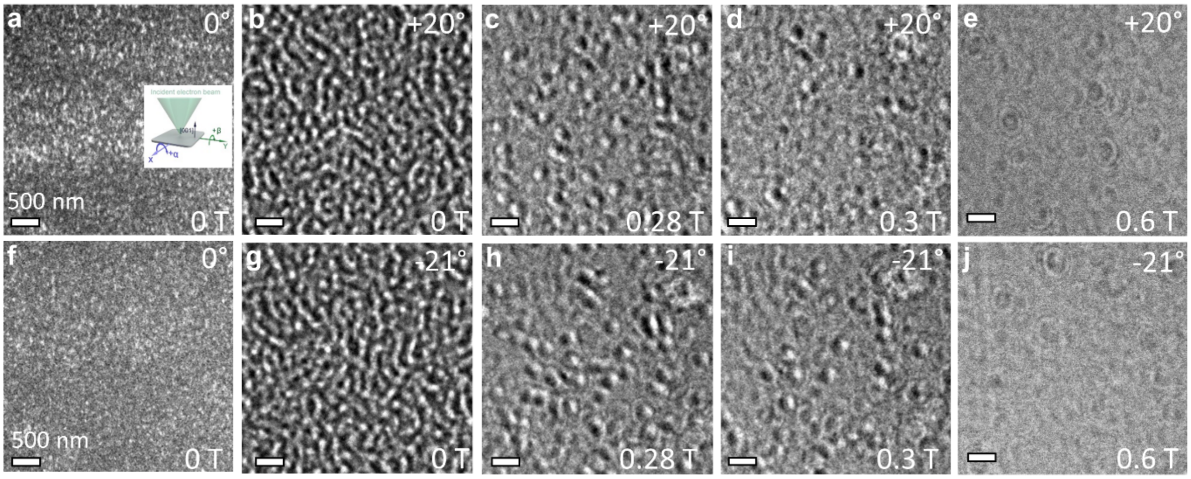

Figure S15: Magnetic field dependent LTEM images of a freestanding membrane formed from 4.3 nm IrAl|27 nm $Co_{2.58}Ni_{0.26}Al$. a-j, LTEM images for various out of plane magnetic fields at 300 K. The LTEM images are acquired at a defocus value of -0.6 mm and with tilting about the *x*-axis of $\alpha = +20^{o}$ and -21° whereas **a**, **f**, are LTEM images acquired in 0 T without any tilt; **b**, **g**, show a cycloidal domain phase under tilting at 0 T; **c**, **d**, and **h**, **i**, show Néel skyrmions under tilting in the presence of out of plane magnetic fields and **e**, **j**, show a field-polarized state under tilting. The inset scale bar corresponds to 500 nm.

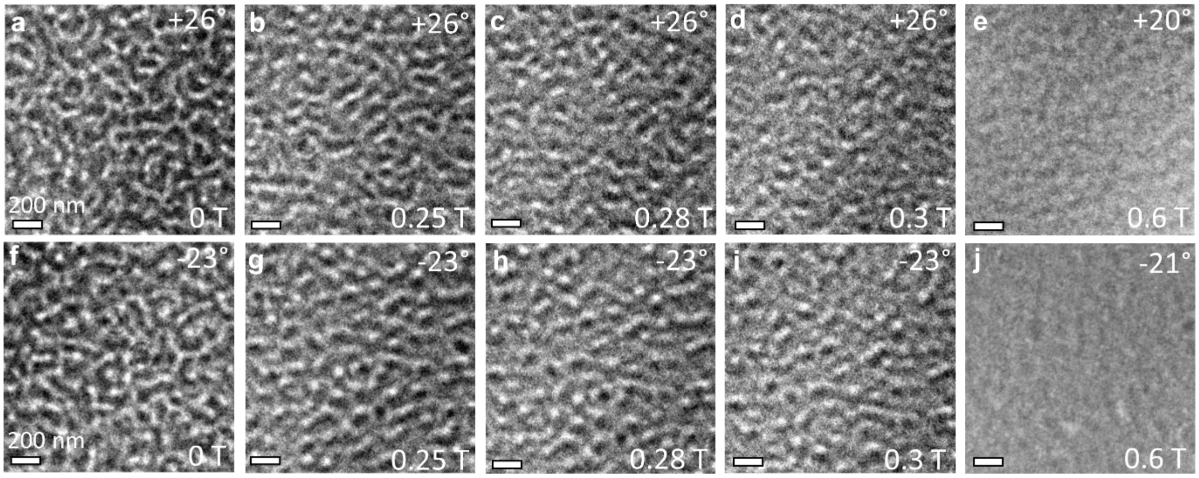

Figure S16: Magnetic field dependent LTEM images in a freestanding bilayer of 4.3 nm IrAl| 27 nm $Co_{2.70}Ni_{0.38}Al$. a-j, LTEM images for various out of plane magnetic fields at 300 K. LTEM images are acquired at a defocus value of -1.0 mm. LTEM images collected with tilting about the *x*-axis: **a-d**, $\alpha = +26^{o}$, **e**, $\alpha = +20^{o}$, **f-i**, $\alpha = -23^{o}$ and **j**, $\alpha = -21^{o}$. **a**, **f**, show a cycloidal domain phase under tilting at 0 T; **b-d**, and **g-i**, show Néel skyrmions under tilting, **e**, **j**, show the field-polarized state. The inset scale bar corresponds to 200 nm.

Superconducting quantum interference device vibrating sample magnetometer (SQUID-VSM) and magneto-transport measurements

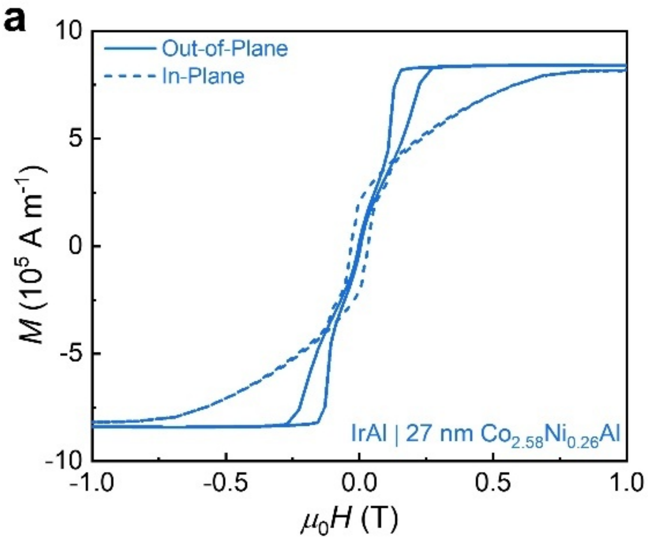

Figure S17: Magnetic hysteresis loops of a 4.3 nm IrAl | 27 nm $Co_{2.58}Ni_{0.26}Al$ thin film structure. a, Out-of-plane and in-plane magnetic hysteresis loops at room temperature.

Properties of 4.3 nm IrAl | *t* $Co_{2.3}Al$ bilayer samples

Bilayer 4.3 nm IrAl | *t* $Co_{2.3}Al$ samples were cut into 3×3 mm^2 chiplets for magnetic characterization. The atomic composition of the IrAl underlayer is $Ir_{42}Al_{58}$ in each case.

Figures S18 and S19 show magnetic hysteresis loops for bilayer 4.3 nm IrAl | *t* $Co_{2.3}Al$ samples for various *t* where the applied magnetic field is oriented perpendicular and parallel to the sample plane, respectively. Figure S20 shows anomalous Hall data for various *t*.

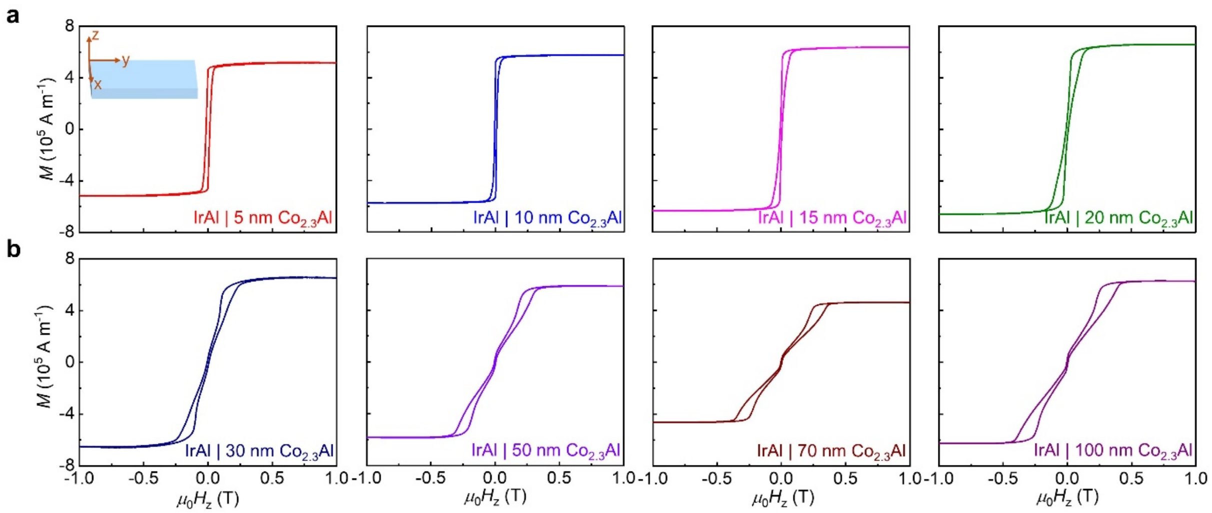

Figure S18: Magnetic properties of IrAl | *t* $Co_{2.3}Al$ bilayer structures. a,**b**, Magnetic hysteresis loops for 4.3 nm IrAl | *t* $Co_{2.3}Al$ bilayers with different thicknesses of the $Co_{2.3}Al$ layers as shown in the inset to the figures. The magnetic field is perpendicular to the sample plane. The inset is a schematic of the magnetization hysteresis loop measurement geometry.

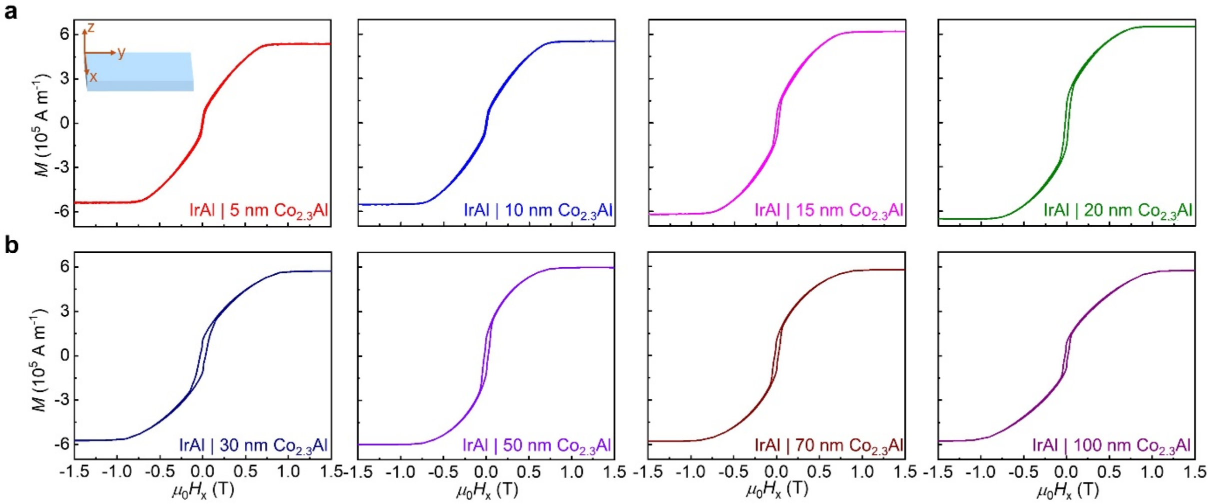

Figure S19: Magnetic properties of IrAl | *t* $Co_{2.3}Al$ bilayer structures. a,**b**, Magnetic hysteresis loops for 4.3 nm IrAl | *t* $Co_{2.3}Al$ bilayers with various thicknesses *t* of the $Co_{2.3}Al$ layers. The field is parallel to the sample plane. The inset figure is a schematic of the magnetization hysteresis loop measurement geometry.

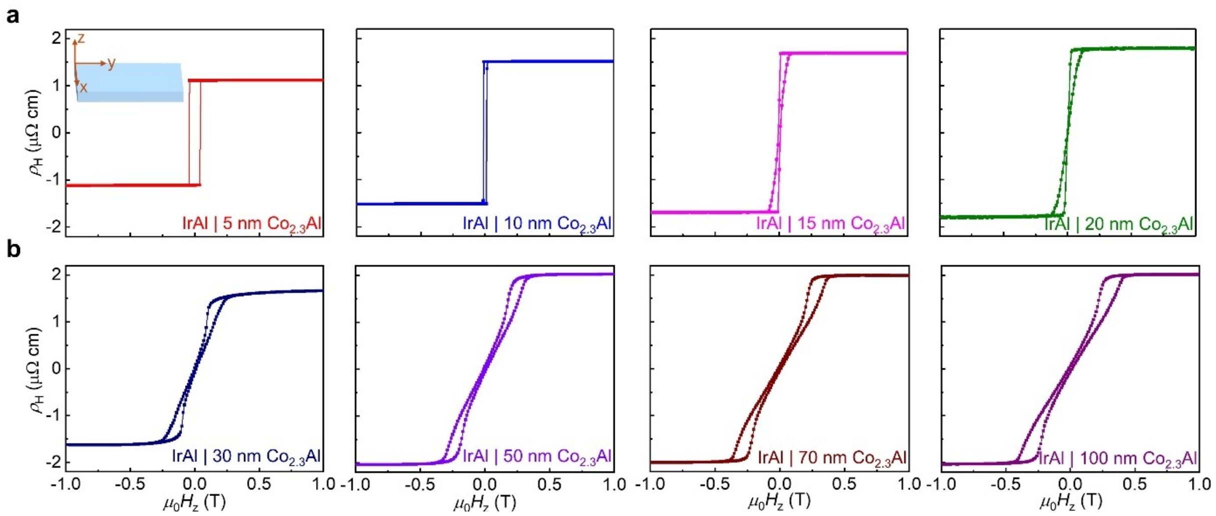

Figure S20: Electrical transport measurements for IrAl | t $Co_{2.3}Al$ bilayer structures. a,**b**, Hall resistivity of 4.3 nm IrAl | t $Co_{2.3}Al$ bilayers with different thicknesses of the $Co_{2.3}Al$ layers. The inset is a schematic of the Hall effect measurement geometry.

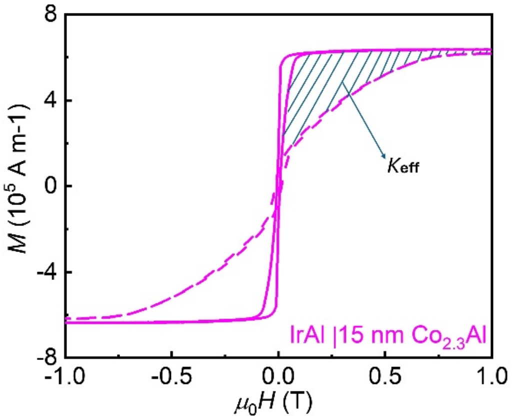

Figure S21: Calculation of effective magneto-crystalline anisotropy (K_{eff}) for 4.3 nm IrAl | t $Co_{2.3}Al$ bilayer structures, e.g. when t = 15 nm.

The saturation magnetization (M_s) from easy-axis, M (H) hysteresis measurements and Hall resistivity (ρ_H) of 4.3 nm IrAl | t $Co_{2.3}Al$ bilayer heterostructure samples obtained at 1T are shown in Figure S22**a** and S22**b**. The effective magnetic crystalline anisotropy of the bilayers, as shown in Figure S22**c**, is calculated based on the equation:

$$K_{\text{eff}} = \frac{M_S H_k}{2} \quad (1)$$

where, H_k is the anisotropy field obtained from the hard axis M (H) loop in Figure S8. We rigorously quantify the K_{eff} by calculating the covered area of out-of-plane magnetization hysteresis loop minus the covered area of in-plane magnetization hysteresis loop in the magnetic field range from 0 to 1 T, as shown in Figure S21.

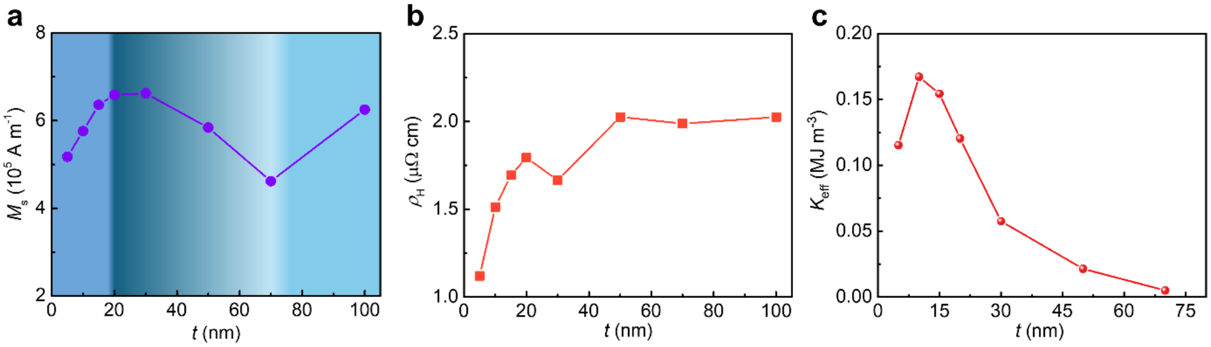

Figure S22: Magnetic and electrical transport properties of 4.3 nm IrAl | *t* $Co_{2.3}Al$ thin film structures **a**, Saturation magnetization, **b**, Hall resistivity and **c**, effective magneto-crystalline anisotropy versus thickness, *t*, of the $Co_{2.3}Al$ layer in 4.3 nm IrAl | *t* $Co_{2.3}Al$ structures.

LTEM analysis of selected samples

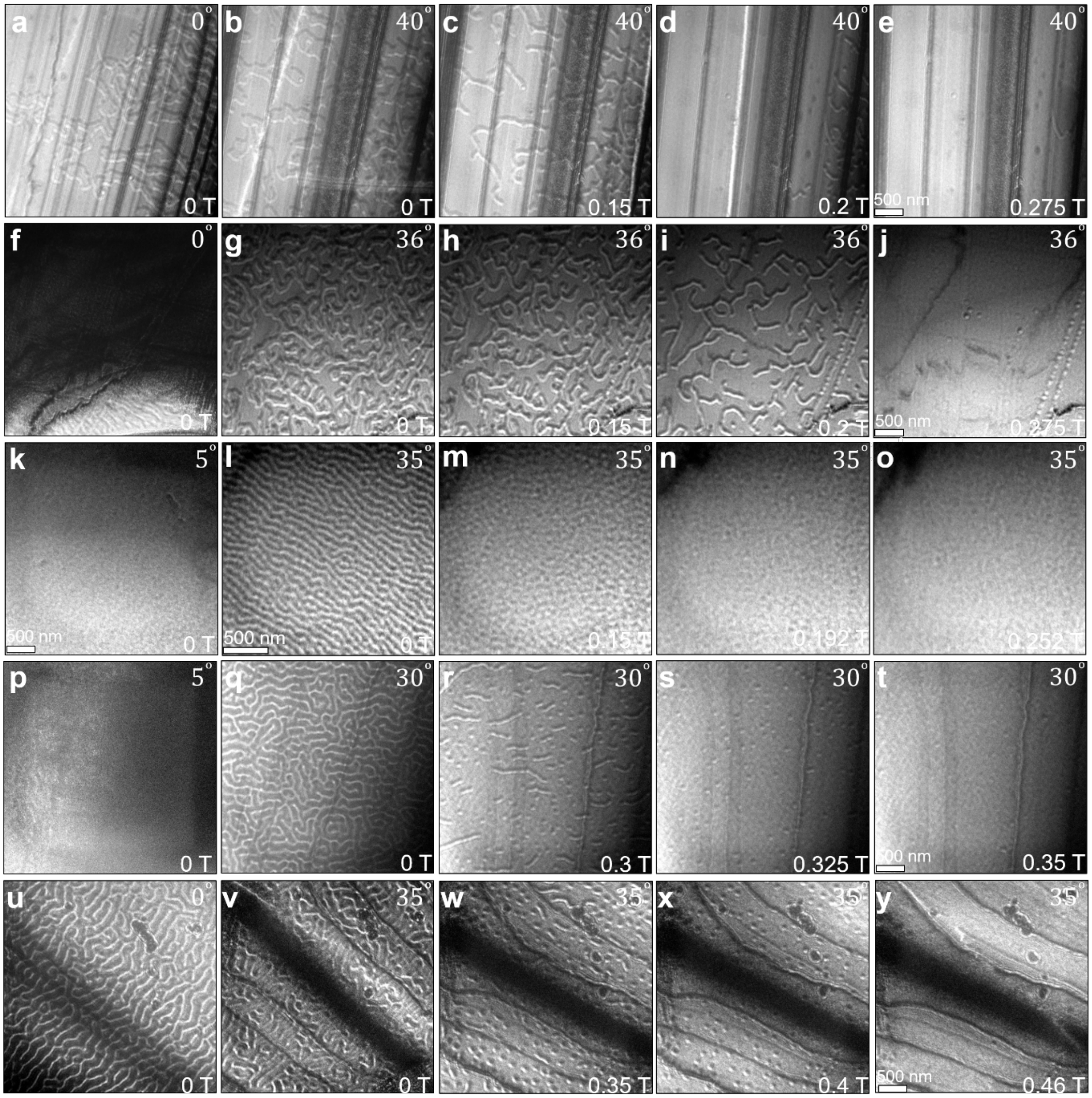

Figure S23: Magnetic field dependent LTEM images for a bilayer of 4.3 nm IrAl| *t* $Co_{2.3}Al$, as a function of *t*. a-y, LTEM images for t = 15 nm **a-e**, 20 nm **f-j**, 30 nm **k-o**, 50 nm **p-t**, and 70 nm **u-y**, for varying out-of-plane magnetic field at 300 K with sample tilting about the x axis. The LTEM images are acquired at a defocus value of -1.5 mm except for **k-o**, that are taken at a defocus value of -1.0 mm. All the samples except samples with t = 30 and 50 nm show stripe domains and bubble phases, while the samples with t = 30 and 50 nm show the presence of a cycloidal state and Néel skyrmions. Note that all images in groups **a-k**, **p-y** and **l-o** are at the same magnification with the scale bar corresponding to 500 nm shown in **k**, **y**, and **I**, respectively.

In Figure S23, we present the magnetic field-dependent LTEM images of 4.3 nm IrAl | *t* $Co_{2.3}Al$ bilayers with varying $Co_{2.3}Al$ thicknesses (*t* = 15, 20, 30, 50, and 70 nm) at room temperature. The tilt angles shown in the images correspond to the total α tilt with respect to the [001] zone axis, rather than the zero position of the TEM holder. For the 15 nm and 20 nm samples, magnetic contrast is observed at zero magnetic field without tilting (**a**, **f**). In Figure S23**b-e** and **g-j**, field-dependent LTEM images under tilt are shown for *t* = 15 and 20 nm, respectively. At 15 nm, only stripe domains appear, with no bubbles observed under applied magnetic fields, and the stripe domains persist up to 0.15 T. At 20 nm thickness, stripe domains coexist with a few type-II magnetic bubbles under magnetic fields, with the stripe domains stable up to 0.2 T.

For bilayers with $Co_{2.3}Al$ thicknesses of 30 nm and 50 nm, no LTEM contrast is observed without tilting. Even after a 5° tilt, no magnetic contrast is visible (Figure S23**k**, **p**). However, upon tilting the 30 nm and 50 nm samples by 35° and 30°, respectively, cycloidal domains emerge at zero field. With increasing magnetic field, these cycloidal domains evolve into Néel-type skyrmions. In the 30 nm film, Néel-type skyrmions remain stable up to 0.2 T, whereas in the 50 nm film they persist up to 0.35 T.

When the $Co_{2.3}Al$ layer thickness in the bilayer is further increased to 70 nm, stripe domains appear at zero field and zero tilt and remain unchanged at a 35° tilt. Under applied magnetic fields, these stripe domains gradually transform into type-II magnetic bubbles (Figure S23**u**–**y**), which remain stable up to a relatively higher field of 0.4 T compared to thinner $Co_{2.3}Al$ bilayer samples.

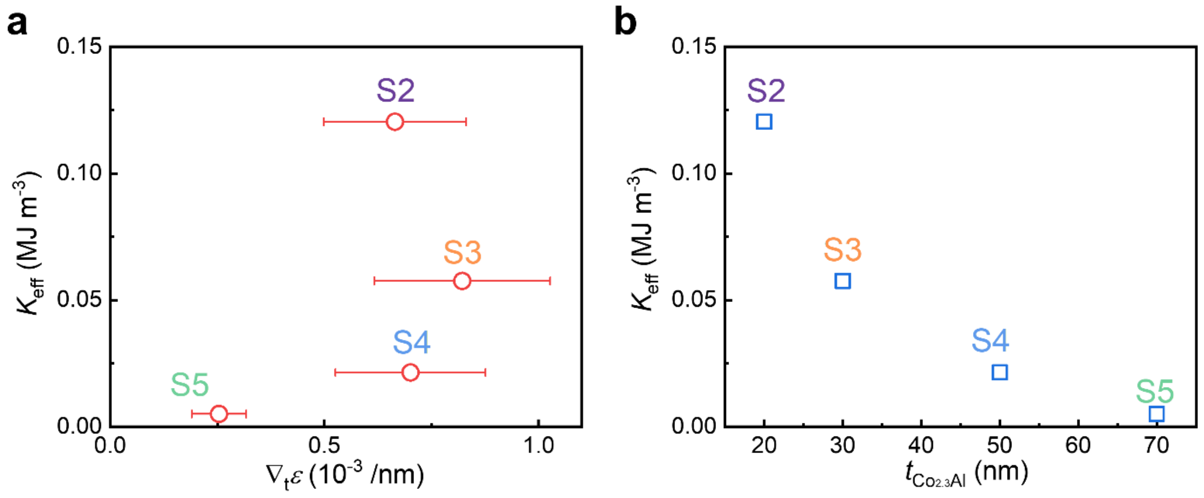

Figure S24: Variation of the effective perpendicular magnetic anisotropy K_{eff} with strain gradient and magnetic layer thickness. a, **b**, K_{eff} versus strain gradient **a**, and thickness **b**, for samples s2 to s5.

Properties of single layer of *t* $Co_{2.3}Al$

The thickness dependent variation of the magnetization, M_s, and anomalous Hall resistivity, ρ_H, versus out-of-plane magnetic field for single layer $Co_{2.3}Al$ films with varying thicknesses at 300 K are shown in Figures S25**a** and S25**c**, respectively. The variation of M_s and ρ_H at 1 T are shown as a function of $Co_{2.3}Al$ layer thickness in Figures S25**b**, and S25**d**, respectively.

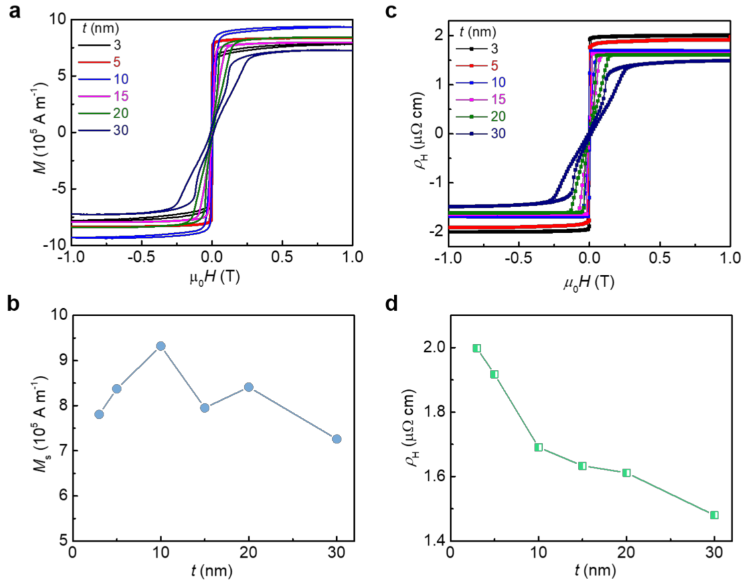

Figure S25: Magnetic and electrical transport properties of $Co_{2.3}Al$ films with various thicknesses. a, Room temperature out-of-plane M(H) loops of $Co_{2.3}Al$ thin films for various thicknesses, t, measured by SQUID-VSM. **b**, Saturation magnetization of $Co_{2.3}Al$ thin films versus thickness, t. **c**, Anomalous Hall measurements of $Co_{2.3}Al$ thin films for various thicknesses, t. **d**, Anomalous Hall resistivity (ρ_H) measured at 1 T for $Co_{2.3}Al$ thin films versus thickness t.

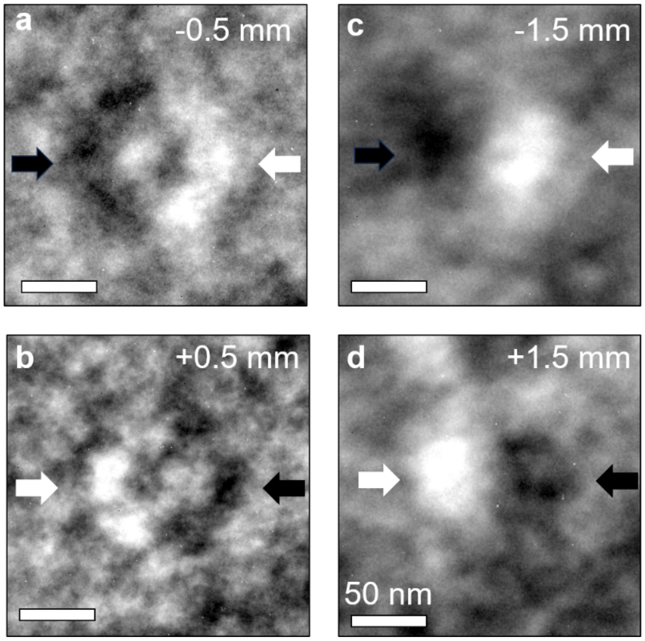

Figure S26: LTEM images of single layer of 30 nm $Co_{2.3}Al$ at 300 K at different defocus distances. a-d, LTEM images of single layer of 30 nm $Co_{2.3}Al$ showing type-II magnetic bubbles for defocus distance of ± 0.5 mm **a**, and **b**, and ±1.5 mm **c**, and **d**, in the presence of an out-of-plane magnetic field 0.26 T under zero tilting. In all these images the scale bar is 50 nm.

In Figure S26, we show the defocus distance dependent LTEM images of MgO|30 nm $Co_{2.3}Al$, under zero tilt in the presence of a 0.26 T magnetic field applied along [001]. At an under-focus value of -0.5 mm (Figure S26**a**) we see a magnetic contrast, which consists of a circular region with a large uniformly magnetized core along with a periphery with dark and bright contrast at their opposite edges. This contrast is similar to the LTEM contrast of a type-II magnetic bubble which is an achiral magnetic texture, that does not require any DMI. Since $Co_{2.3}Al$ is centrosymmetric in the absence of any strain gradient (i.e., without the IrAl underlayer) there is no DMI. The magnetic contrast reverses while collecting images under a defocus distance of +0.5 mm, as shown in Figure S26**b**. However, we see that while recording the LTEM images of the same magnetic textures at a higher defocus distance (±1.5 mm), there is a distortion of the magnetic

contrast, where the central uniformly magnetized region is not visible, but rather the boundary region with dark and bright contrast now becomes more prominent, as shown in Figures S26**c** and S26**d**. The magnetic contrast at the larger defocus (±1.5 mm) looks more like a Néel-type skyrmion, as discussed with reference to the previous figure, although the texture is a type-II magnetic bubble. Thus, the real nature of a magnetic texture can only be inferred from LTEM imaging at a lower defocus value, until it shows any sign of distortion at a larger defocus value. Therefore, the defocus distance dependent LTEM images are very important to distinguish the type of the magnetic texture.

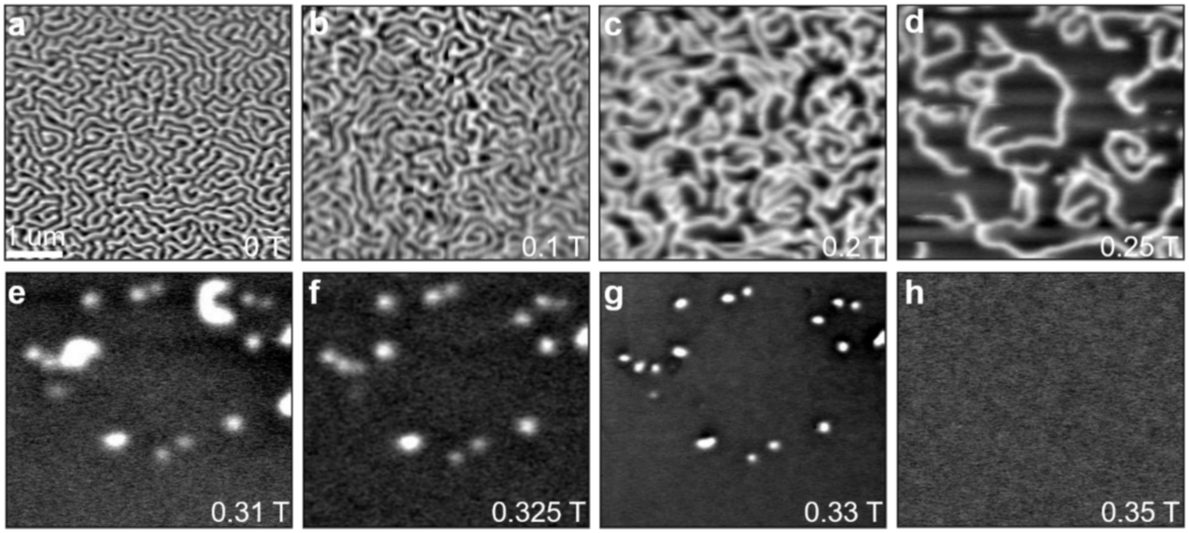

Figure S27: Magnetic force microscopy (MFM) images of single layer of $Co_{2.3}Al$ at 300 K. a-h, Magnetic force microscopy (MFM) images of 50 nm $Co_{2.3}Al$ films directly grown on a MgO(001) substrate with 2 nm MgO buffer layer, showing evolution of the spin textures with varying magnitude of the out-of-plane magnetic field. The scale bar for all MFM images is 1 µm.

All MFM experiments were carried out in an Attocube liquid helium cryostat system equipped with a 2D vector superconducting magnet under UHV conditions. A magnetic tip from Nanosensors (SSS-MFMR) was used for all measurements. The soft coating on the magnetic tip ensures a minimal disturbance of the magnetic state of the samples (low moment tip for non-perturbative imaging with high spatial resolution). The high mechanical quality factor ($Q > 30000$) accounts for high force sensitivity. The tip was magnetized in a negative z direction by a permanent magnet.

Dual scanning comprised of topographic and magnetic scans was used to separate atomic and magnetic forces. The tip first interacts with the sample in tapping mode and the surface topography is recorded. Subsequently, the tip is lifted by 60-80 nm above the sample surface during the second scan to enable imaging of the magnetic interactions. Great care was taken to eliminate tip induced artefacts and perturbations. An out-of-plane magnetic field was applied perpendicular to the sample surface to obtain images of the magnetic spin textures. The cantilever oscillates at its resonant frequency, which is modified by its interaction with the sample's stray field from any magnetic force gradient. The frequency shift (Δf) can be detected in one of the following ways: phase modulation and frequency modulation. We use phase detection owing to its large signal-to-noise ratio and ease of use.

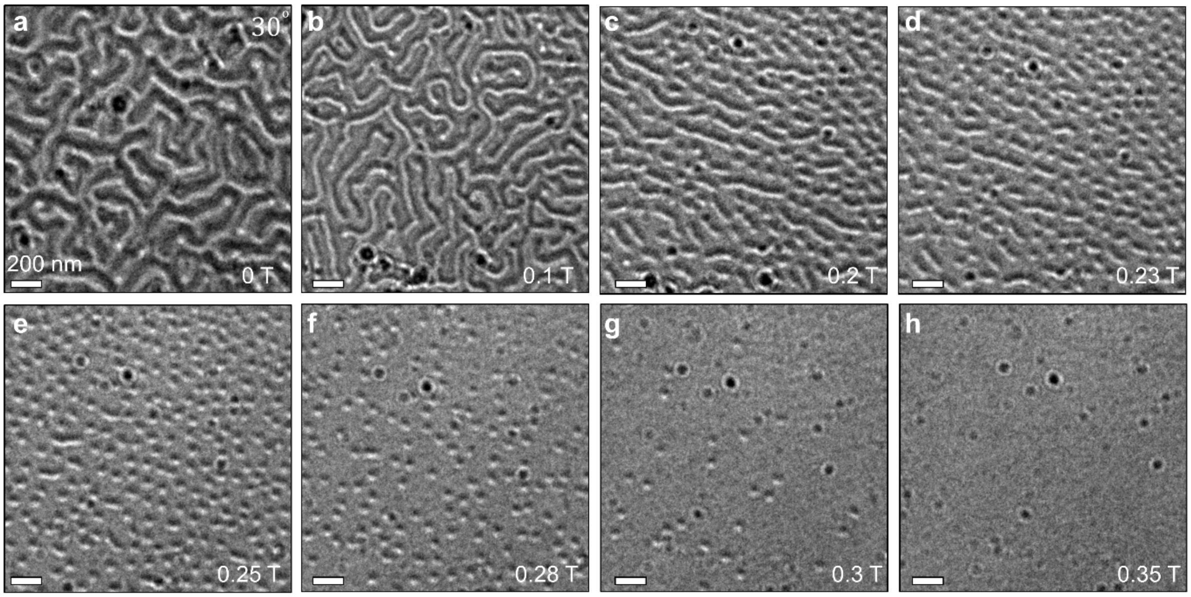

Figure S28: Magnetic field dependent LTEM images for 4.3 nm IrAl | 0.3 nm Al | 30 nm $Co_{2.3}Al$. a-h, LTEM images recorded for varying out of plane magnetic field at 300 K showing the evolution of Néel skyrmions. The LTEM images are acquired at a defocus value of -1.0 mm and under tilting about the *x*-axis, α = 30°. The scale bar corresponds to 200 nm.

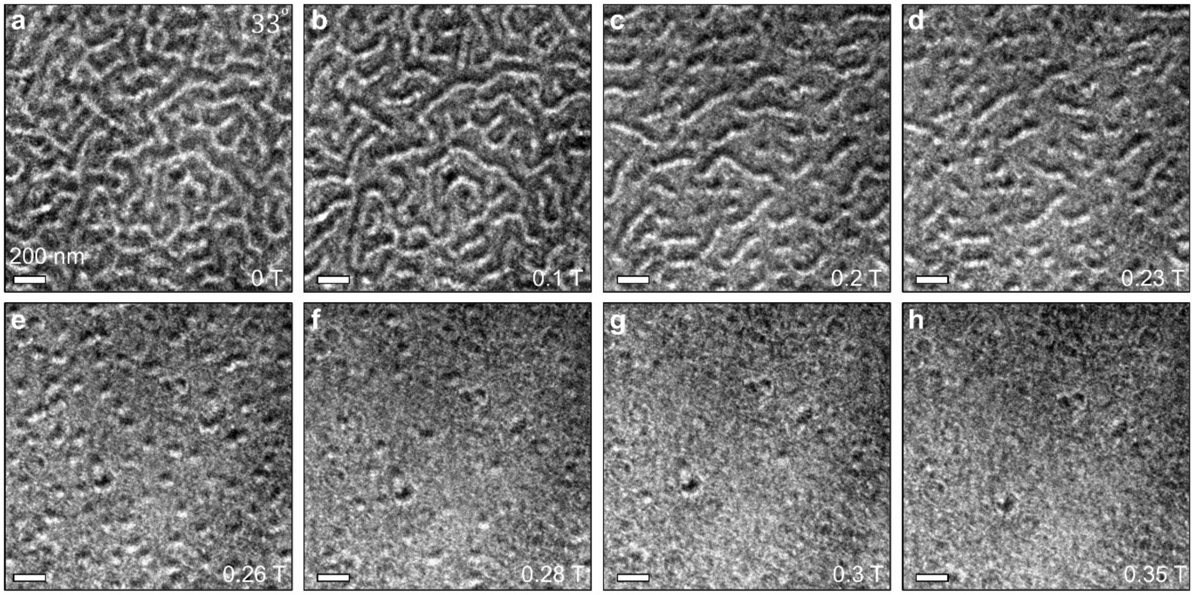

Figure S29: Magnetic field dependent LTEM images for 4.3 nm IrAl | 30 nm $Co_{2.3}$Al | 2 nm IrAl. a-h, LTEM images under a varying magnetic field at 300 K showing the transition from cycloidal phase to Néel skyrmions. The LTEM images are acquired at a defocus value of -1.0 mm and under tilting about the *x*-axis, α = 33°. The scale bar corresponds to 200 nm.

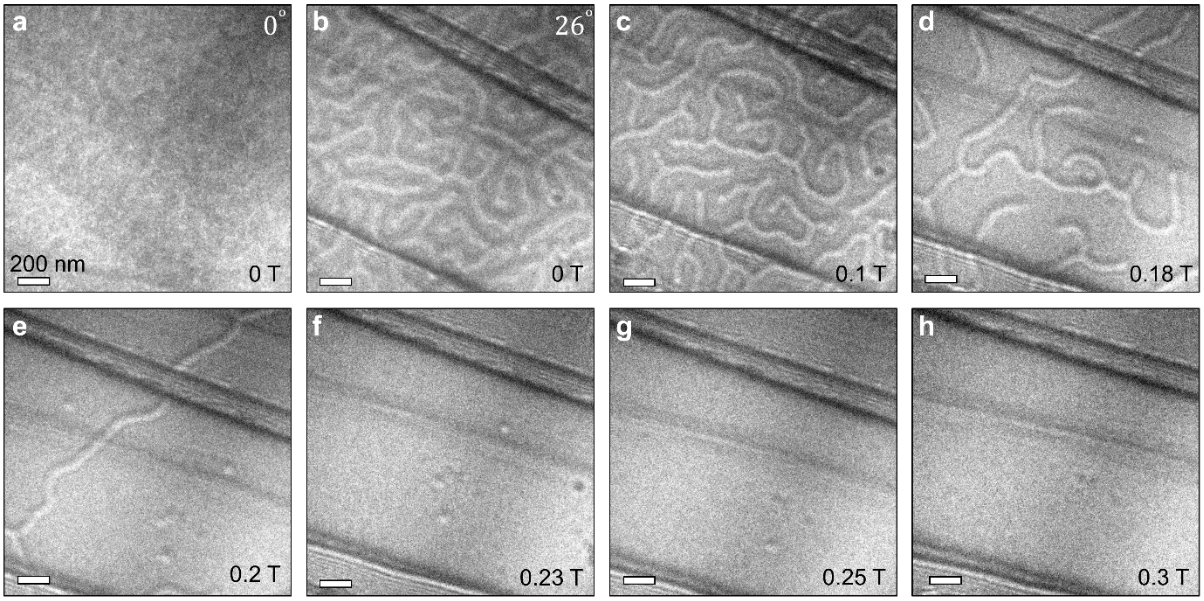

Figure S30: Magnetic field dependent LTEM images for 1 nm IrAl|30 nm $Co_{2.3}$Al. a-h, LTEM images under a varying magnetic field at 300 K with tilting about the *x*-axis, α = 0° **a**, α = 26° **b-h** showing only stripes. All LTEM images are acquired at a defocus value of -1.0 mm. The scale bar corresponds to 200 nm.

Properties of single layer of Co$_x$Al samples

The 10×10 mm^2 single layer Co$_x$Al thin films (without any IrAl underlayer, only 2 nm MgO buffer layer underneath) capped with 4 nm thick MgO as protective layer were cut into pieces with a size of 2.5×5.5 mm^2 for SQUID-VSM (Superconducting Quantum Interference Device - Vibrating Sample Magnetometer) measurements. The magnetic field is oriented along the direction perpendicular to the sample plane. Figure S31**a** shows magnetic hysteresis loops for several Co$_x$Al thin films with different compositions. The saturation magnetization (M_s) of the Co$_x$Al thin films was estimated at around 0.6 T. The variation of M_s as a function of the composition (x) and out-of-plane lattice parameter (c) is shown in Figures S31**b** and S31**c**, respectively.

The sheet resistance (R_{sq}) of the Co$_x$Al alloy thin films was measured with a four-point in-line probe method. A direct current source (Keithley 6221), and a nanovoltmeter (Keithley 2182a) were used for the Hall measurements. The sheet resistivity ρ is obtained from, $\rho=\delta\times R_{sq}\times t$, where t is the thickness of the film. The coefficient δ depends on the sample dimensions and the length-to-width ratio (l/w). In here, δ is 0.7744 according to the ratio between sample width ($l=w=10$ mm) to four-point probe spacing ($s=1.59$ mm)[4]. The variation of ρ as a function of x is shown in Fig. S31**b**. The Hall resistivity was measured using the van der Pauw method.

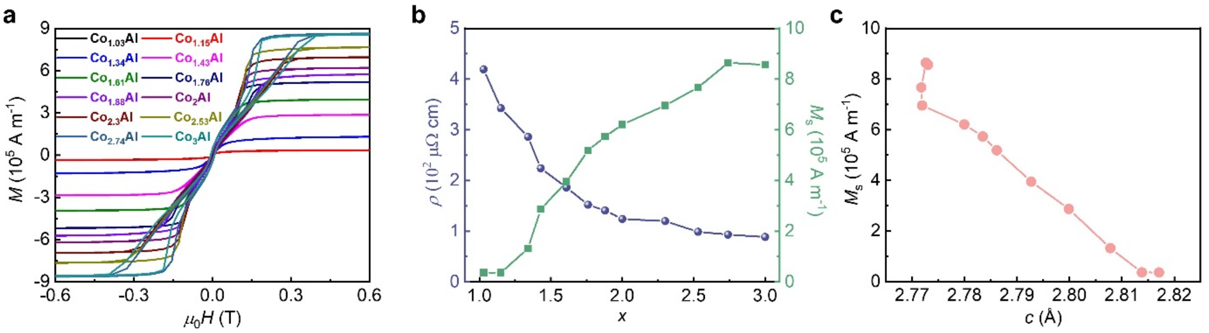

Figure S31: Magnetic and electrical transport properties of Co$_x$Al alloy films. a, Magnetic hysteresis loops M (H) of several Co$_x$Al alloy thin films as a function of out-of-plane magnetic field measured by SQUID-VSM. **b**, Sheet resistivity (ρ) and saturation magnetization (M_s) of the Co$_x$Al alloy films as a function of x. **c**, Plot of saturation magnetization versus out-of-plane lattice parameter c of Co$_x$Al alloy thin films.

Properties of bilayer 4.3 nm IrAl | 30 nm Co_xAl (x = 2.0, 2.3, 2.6, and 2.9) samples

The samples were cut into 3×3 mm^2 chiplets for magnetization measurements. The magnetization hysteresis loops, shown in Figure S32**a**, were recorded with a magnetic field applied perpendicular to the sample plane. The saturation magnetization (M_S) derived from the M (H) loops at 0.75 T versus the Co concentration (x) in Co_xAl is shown in Figure S33**b**. The measurements of the Hall resistivity were carried out using the same samples and are shown in Figure S32**c**. The Hall resistivity obtained at 0.75 T is plotted as a function of x in Figure S32**d**.

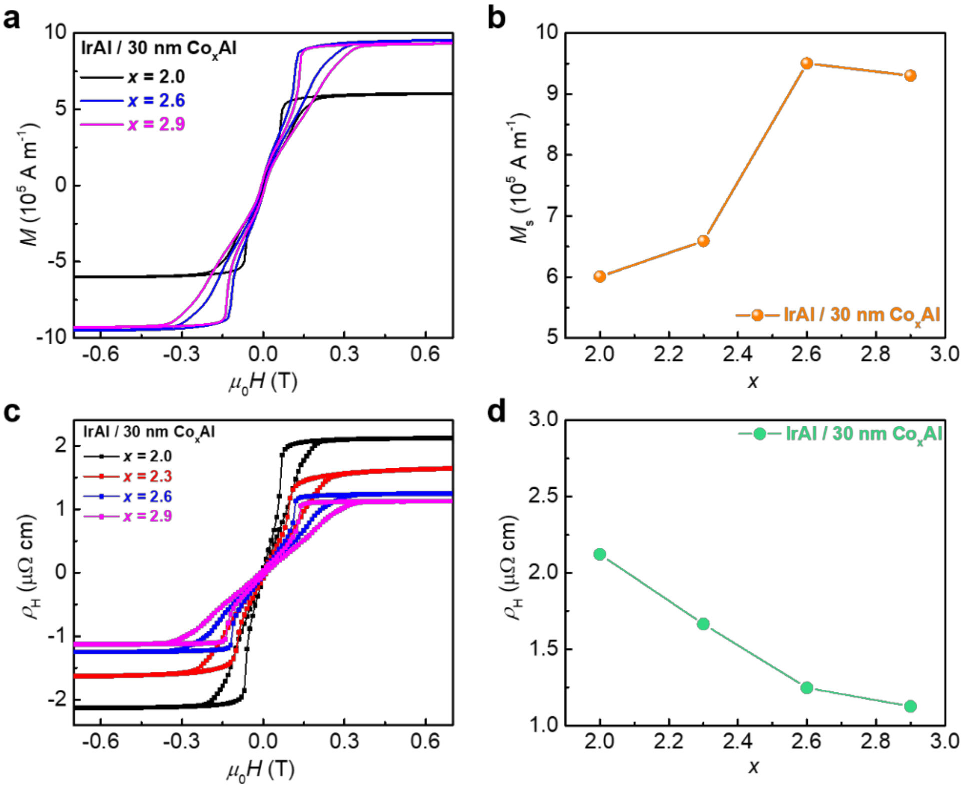

Figure S32: Magnetic and electrical properties of 4.3 nm IrAl | 30 nm Co_xAl bilayer structures. a, Magnetic hysteresis loops for 4.3 nm IrAl | 30 nm Co_xAl bilayers of different compositions (x) of cobalt measured using a SQUID-VSM with field perpendicular to the sample plane. **b**, Saturation magnetization (M_S) versus x. **c**, Field dependent Hall resistivity (ρ_H) for various x. **d**, Hall resistivity obtained at 0.75 T versus x.

Properties of bilayer of 4.3 nm MAl | 30 nm $Co_{2.3}Al$ samples (M= Ru and Pd)

The growth method for 4.3 nm MAl (M= Ru and Pd) | 30 nm $Co_{2.3}Al$ is outlined in the methods section of the main text. The concentration of the heavy metal elements (Pd and Ru) can be tuned by changing the applied sputtering power applied to the corresponding target, while for the aluminum target the sputtering power was kept constant at 95 W. The Hall resistivity (ρ_H) data as a function out-of-plane magnetic field for two films are shown in Figure S33.

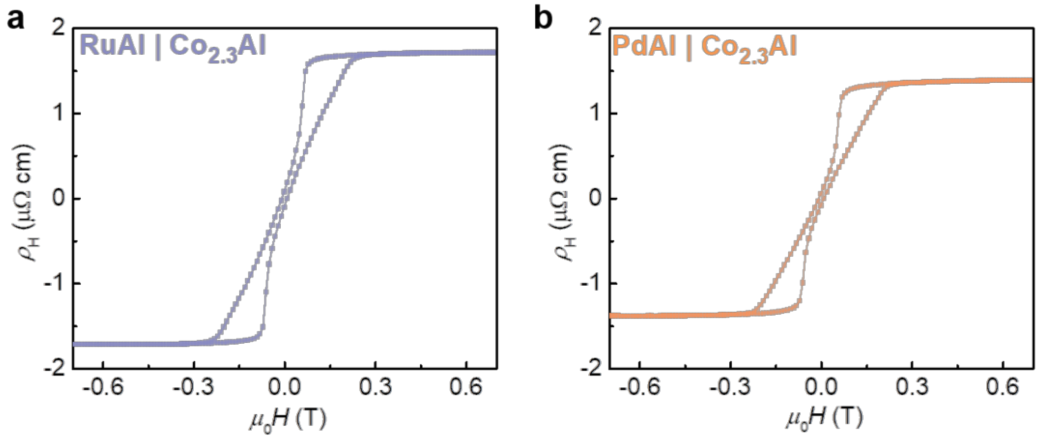

Figure S33: Hall resistivity (ρ_H) of MAl | 30 nm $Co_{2.3}Al$ bilayers. a, Field dependent Hall resistivity for RuAl | 30 nm $Co_{2.3}Al$ and **b**, PdAl | 30 nm $Co_{2.3}Al$, respectively. The atomic composition of the underlayers is $Ru_{46}Al_{54}$ and $Pd_{35}Al_{65}$, and 4.3 nm, respectively. The thickness of each of the underlayers is 4.3 nm.

In Figure S34, we show magnetic field-dependent LTEM images of bilayers of 4.3 nm IrAl | 30 nm Co_xAl at room temperature for x=2.0 (**a-e**), 2.6 (**f-j**) and 2.9 (**k-o**). In all samples, stripe domains are observed at zero magnetic field and under zero tilting. Further LTEM images upon application of magnetic field are shown in the presence of sample tilting about the x-axis as labelled in the figure. With increasing magnetic field, the magnetic stripe phase majorly transforms into field-polarized state along with a few isolated magnetic bubbles in the presence of intermediate magnetic field, but no Néel skyrmions are observed under any condition. Thus, we find that the composition of the Co_xAl layer is critically important to observe Néel skyrmions which we only find for x ~2.3.

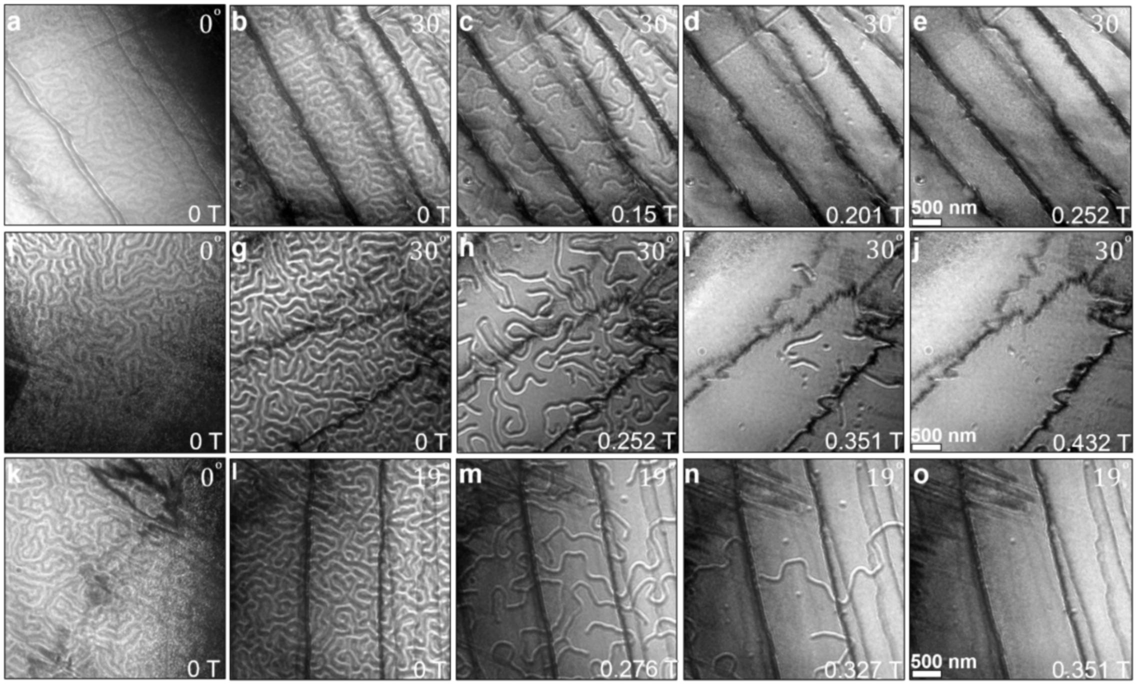

Figure S34: Magnetic field dependent LTEM images for bilayers of 4.3 nm IrAl | 30 nm Co$_x$Al, with varying cobalt composition (x). a-o, LTEM images of a bilayer of 4.3 nm IrAl **|** 30 nm Co$_x$Al, for x = 2.0 **a-e**, 2.6 **f-j**, and 2.9 **k-o**, with varying magnetic field at 300 K. LTEM images are acquired at a defocus value of -1.5 mm. All the images show stripes and bubble phases. The scale bar is 500 nm.

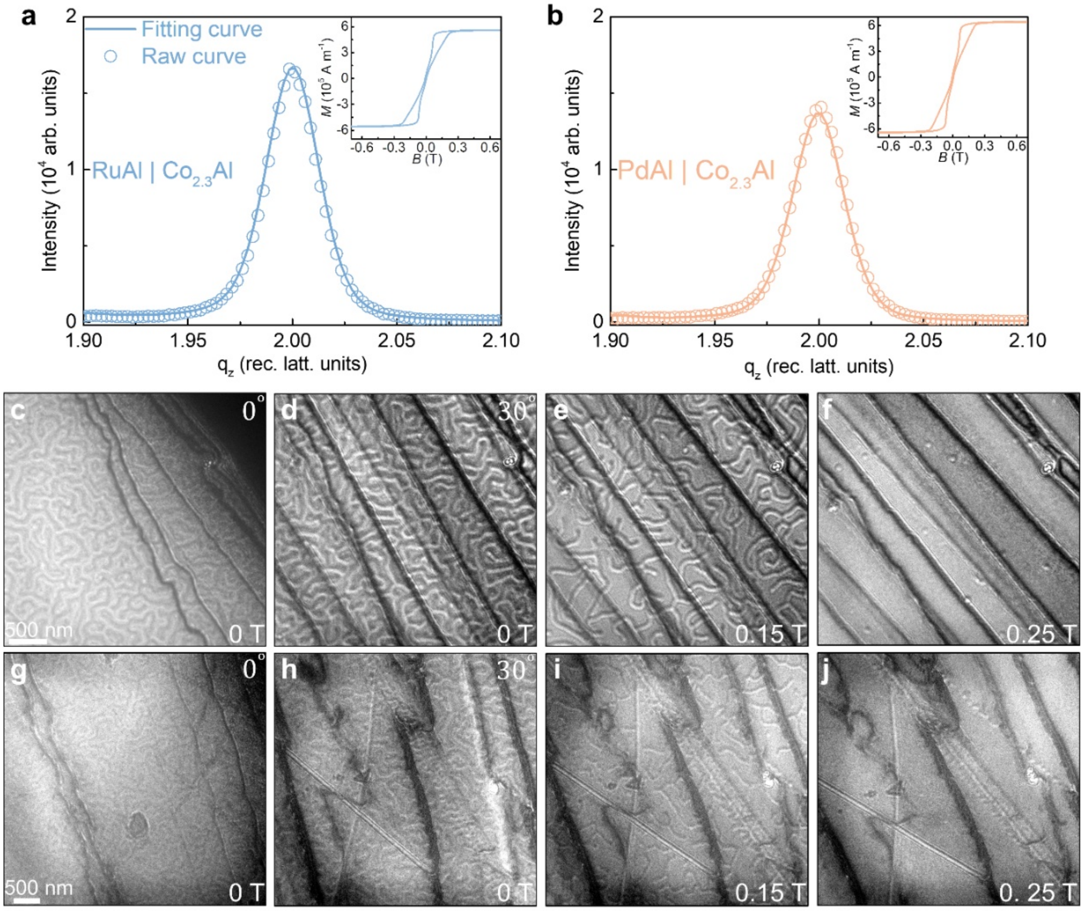

Figure S35: X-ray diffraction analysis, magnetization hysteresis loops and magnetic field dependent LTEM images of bilayers of 4.3 nm MAl (M= Ru and Pd) | 30 nm $Co_{2.3}Al$ at 300 K. **a**,**b**, Analysis of the shape of the X-ray peak in **a** and **b** shows no evidence of any significant strain gradient. The insets show isothermal magnetization data in the presence of an out-of-plane magnetic field at 300 K. **c**-**j**, LTEM images collected at 300 K under varying magnetic fields as labelled and at α = 30° and -1.5 mm defocus (**g**, was recorded at a defocus of -2 mm), **c**-**f**, RuAl underlayer; and **g**-**j**, PdAl underlayer. All the samples show stripe domains and type-II bubble phases. The scale bar for all these images from **c** to **j** is 500 nm.

In Figure S35, we show the x-ray diffraction profile analysis and LTEM images of bilayers of 4.3 nm MAl | 30 nm $Co_{2.3}Al$ containing different underlayers corresponding to M=Ru and Pd. From Figures S35**a** and **b**, it is evident that the (002) XRD peak has a symmetric shape in contrast to those recorded from $Co_{2.3}Al$ with an IrAl underlayer. This directly suggests that there is no significant strain gradient, i.e., $\nabla_t \varepsilon$ is below 10^{-4} /nm range for the samples with RuAl and PdAl underlayers. In Figures S35**c**-**f** and **g**-**j**, we show the corresponding magnetic-field dependent LTEM images from these samples. In both samples, only stripe domains in zero magnetic field in

the absence of any sample tilting are observed. Magnetic field-dependent LTEM images with sample tilting (by 30°) show that application of magnetic field results in the transformation of the stripe phase into a field-polarized state, but skyrmions are not observed under any conditions.

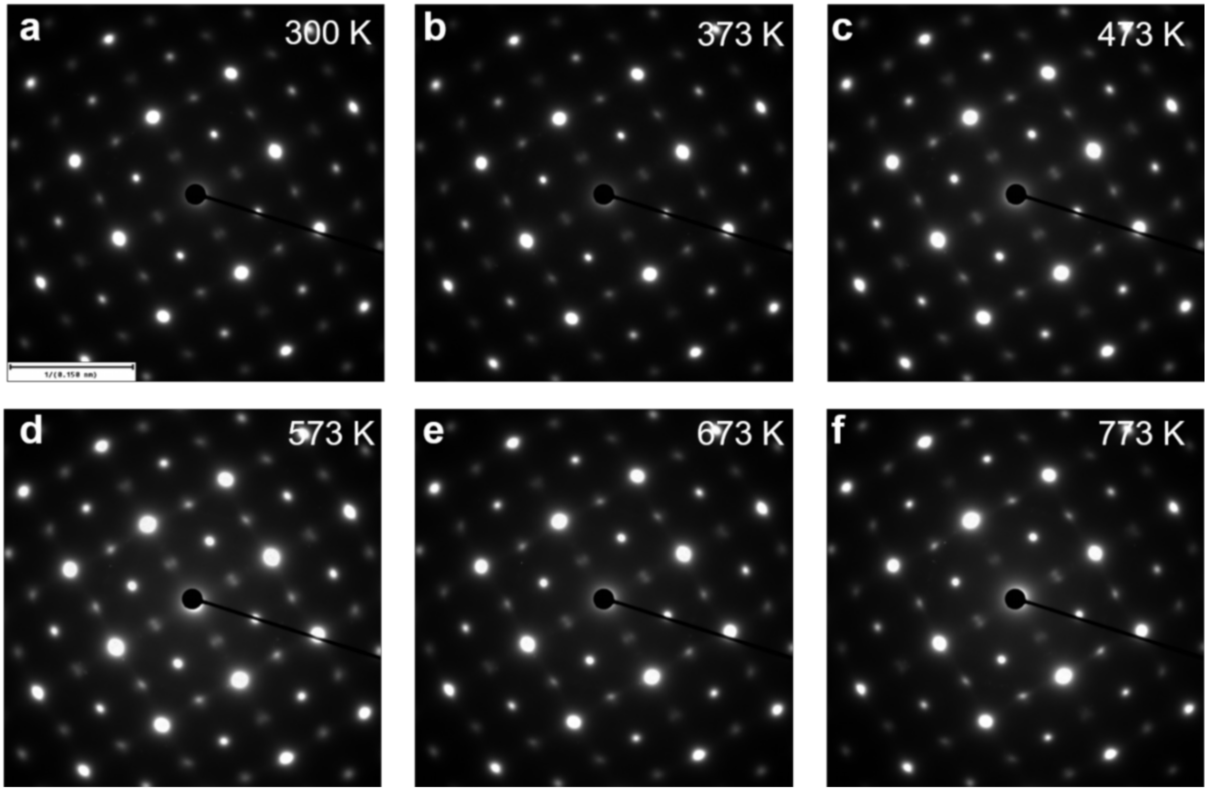

Figure S36: Selected area electron diffraction (SAED) of a freestanding bilayer of 4.3 nm IrAl | 30 nm $Co_{2.3}Al$ under in-situ heating. **a-f**, SAED pattern recorded while heating the sample in-situ shows that the crystalline structure is stable up to the maximum temperature of 773 K.

The SAED patterns of a freestanding bilayer of 4.3 nm IrAl **|** 30 nm $Co_{2.3}Al$ (Figure S36) remain unchanged across all temperatures, confirming that the crystal structure is preserved upon heating. This indicates that the bulk DMI, originating from the strain gradient, is maintained in the freestanding lamella from room temperature to high temperature up to 773 K. Consequently, the emergence of magnetic textures is an inherent effect of the strain-gradient-induced DMI, validating that the LTEM images reflect intrinsic properties of the 4.3 nm IrAl **|** 30 nm $Co_{2.3}Al$ bilayer freestanding membrane.

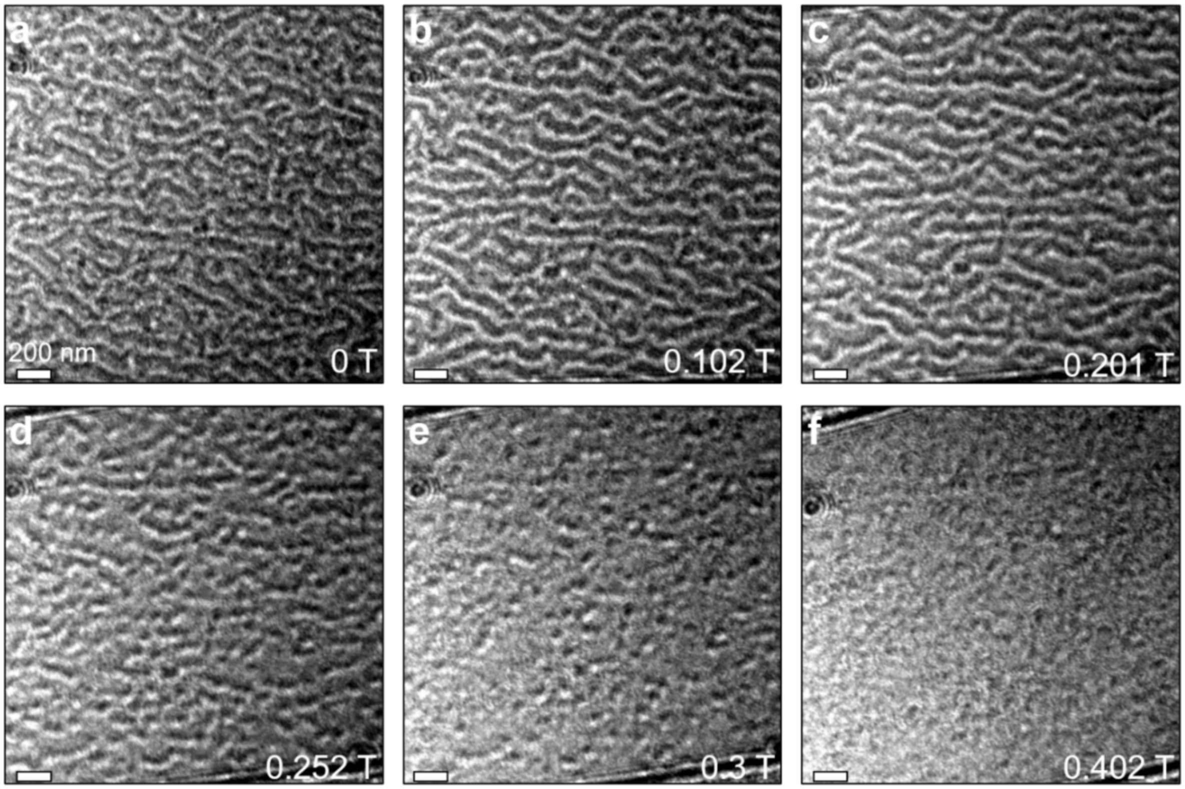

Figure S37: LTEM images of a freestanding bilayer of 4.3 nm IrAl | 30 nm $Co_{2.3}$Al recorded at 300 K. **a-f**, LTEM images recorded with varying magnetic field (see labels) at α = 20° tilt angle showing the transition from labyrinth-type domains to Néel skyrmions above 0.25 T. All LTEM images were taken at a defocus distance of -1 mm. Scale bar is 200 nm.

The LTEM measurements on the freestanding 4.3 nm IrAl | 30 nm $Co_{2.3}$Al bilayer at 300 K reveal a distinct field-driven evolution of the magnetic textures. At zero magnetic fields, cycloidal-type domain structures are observed at a tilt angle of 20°, which progressively transform into Néel-type skyrmions as the field increases beyond 0.25 T. These results demonstrate the robust stabilization of skyrmions in the freestanding bilayer, highlighting the influence of the strain gradient inherited from the IrAl underlayer and confirming that the effect is intrinsic.

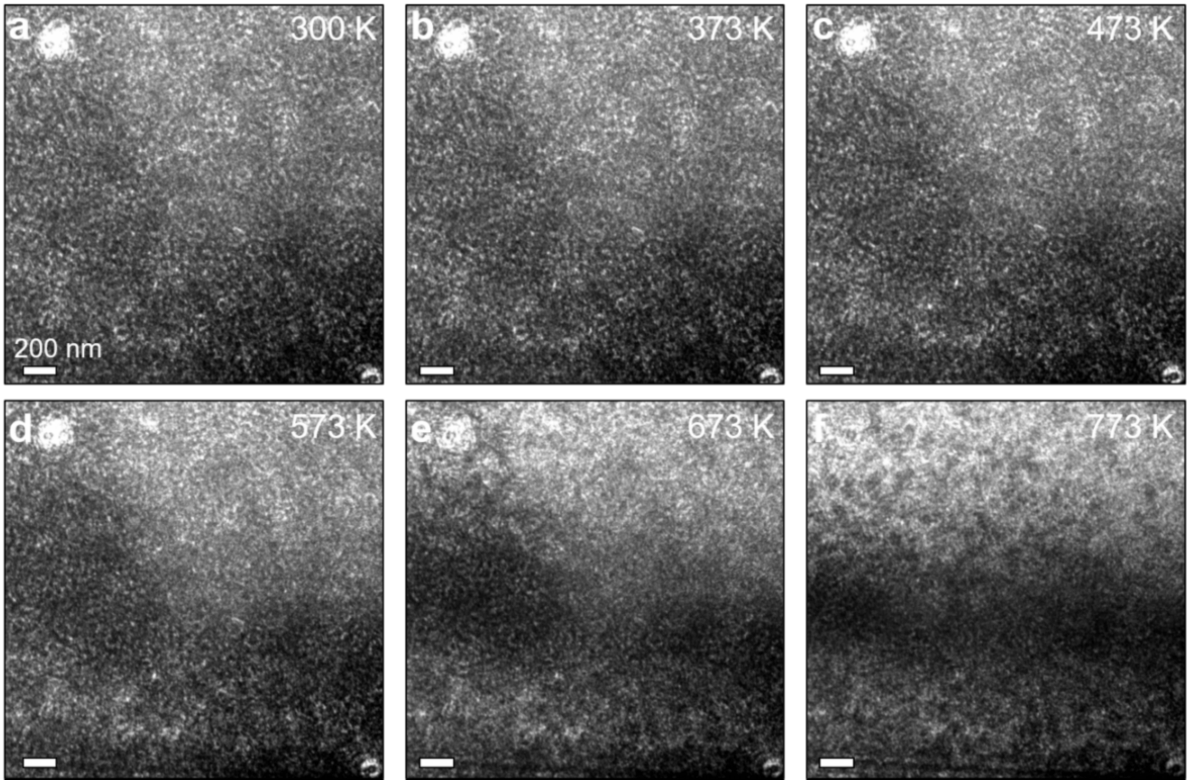

Figure S38: LTEM images of a freestanding bilayer of 4.3 nm IrAl | 30 nm $Co_{2.3}Al$ recorded under in-situ heating. **a-f**, LTEM images recorded in zero magnetic field at $\alpha = 0°$. No magnetic contrast is observed. All LTEM images are taken at a defocus distance of -1 mm. Scale bar is 200 nm.

The LTEM measurements on the freestanding 4.3 nm IrAl **|** 30 nm $Co_{2.3}Al$ bilayer under in-situ heating show no magnetic contrast at $\alpha = 0°$ in zero field, confirming that tilt is required to reveal domain structures, as demonstrated in previous section (Figure S37). This absence of contrast is consistently observed even at elevated temperatures (Figures S38**b**–**f**)

Modelling of the strain gradient

We model a CoAl film on an IrAl substrate. In-plane lattice constants *a* and *b* are taken from bulk IrAl and are fixed for the substrate and through the entire film (a=b=2.7 Å). To model a strain gradient in the film, we introduce the lattice constant $c(t)$, where t is the film thickness. Therewith, $c\ (0) = c_0$=2.7 Å is the lattice constant c of the IrAl substrate. At the top of the film L, the lattice constant $c(L) = c_L$=2.933 Å, the lattice constant c of the tetragonal CoAl alloy. In our simulations, we model the following situation where:

$$c(t) = \ c_0 + At,\ \ t \in [0, L] \tag{2}$$

i.e., the lattice constant c increases linearly in t such that A is given by:

$$A\ = \frac{c_L - c_0}{L} \tag{3}$$

Stress is defined as the ratio:

$$\varepsilon_t = \frac{c(t) - c_L}{c_L} = \frac{c_0 + At - c_L}{c_L} \tag{4}$$

The strain gradient is given by:

$$\nabla_t \varepsilon = \frac{\partial \varepsilon_t}{\partial t} = \frac{A}{c_L} \tag{5}$$

In our first-principles calculations, the film is divided into N unit cells along the film normal, whose lattice constants are given by:

$$c_i = \ c_0 + w(i-1),\ i\ \in\ [1, N],\ \ \ w = \frac{c_L - c_0}{N-1} \tag{6}$$

From the above equations, we deduce:

$$w = A\ \frac{L}{N-1} = \frac{c_L L}{N-1} \nabla_t \varepsilon \tag{7}$$

The film thickness is given by:

$$L = \ \sum_{i=1}^{N} c_i = \ \frac{N}{2}\ (c_0 + c_L) \tag{8}$$

Altogether, we find that

$$w = \ \frac{c_L \frac{N}{2}(c_0 + c_L)}{N-1} \nabla_t \varepsilon\ \approx\ \frac{1}{2}\ c_L(c_0 + c_L)\nabla_t \varepsilon \tag{9}$$

The last approximation is justified as the number of unit cells along the film normal is much larger than 1.

In our case, c_0 = 2.7 Å and c_L = 2.933 Å, such that $w \approx 8.26$ Å^2 $\nabla_t \varepsilon$

Micromagnetic simulations

Topological charge

The topological charge density is defined as[5]:

$$\rho_T(\boldsymbol{r}) = \frac{1}{4\pi}\,\boldsymbol{m} \cdot \left(\frac{\partial \boldsymbol{m}}{\partial x} \times \frac{\partial \boldsymbol{m}}{\partial y}\right) \quad (10)$$

The total topological charge Q is thus given by:

$$Q = \int_S \mathrm{d}A\, \rho_T(\boldsymbol{r}), \qquad A = \pi r^2 \qquad (11)$$

where S is the two-dimensional area under investigation.

Miscellaneous

All figures showing the results of micromagnetic simulations were produced using MuView[6] and Matplotlib[7].

Results for $\nabla_t \varepsilon = 0.003$ /nm

The magnetic field is varied in ten steps between $\mu_0 H_z = 0$ T and $\mu_0 H_z = 1$ T. The mesh size is 512×512×8 cells, i.e., we define eight regions stacked along the film normal. D varies between 0.2 mJ/m^2 and 3.1 mJ/m^2, $A \approx 11.7$ pJ/m, $M_S \approx 650$ kA/m and $K_{\mathrm{eff}} \approx 1.09 \times 10^5$ J/m^3. The results are presented in Figure S40. In the absence of an external magnetic field, a labyrinth-like state is observed. Upon an increase of the external field, skyrmions of topological charge Q = -1 and magnetic stripes become stable. Further amplification of the magnetic field shrinks the structures and ultimately leads to a fully polarized state. Within our simulations, the skyrmions' radii show only a negligible z-dependence.

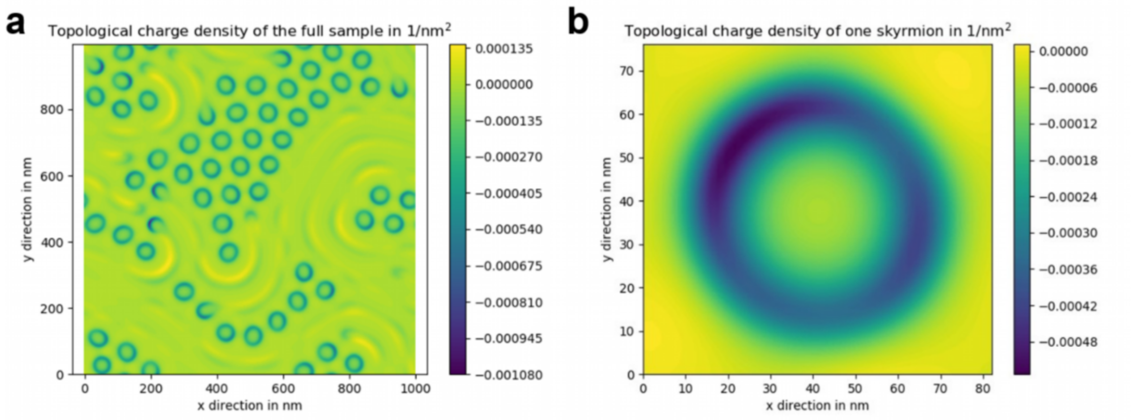

Figure S39: a, Topological charge density of the total simulated sample and **b**, a Néel-type skyrmion, both at $\mu_0 H_z$ = 0.2 T and $\nabla_t \varepsilon$ = 0.003 /nm.

Modelling the uniaxial anisotropy contribution

The micromagnetic simulations assume that the uniaxial anisotropy, whose magnitude has been determined experimentally, is constant within the film. To test this assumption, this homogeneity was broken in two ways. First, we assumed that the anisotropy might have a linear dependence on the distance from the substrate, increasing from the bottom to the top of the film as represented by the black curve in Figure S40. Second, we assumed that the anisotropy to be stronger at the vertical boundaries of the film, which was modelled through a potential function which mimics a strong increase of the anisotropy at both interfaces (See Figure S40). In both cases, the anisotropy was chosen so that the average value matches that of the original simulation. Figure S40 depicts the choice of parameters.

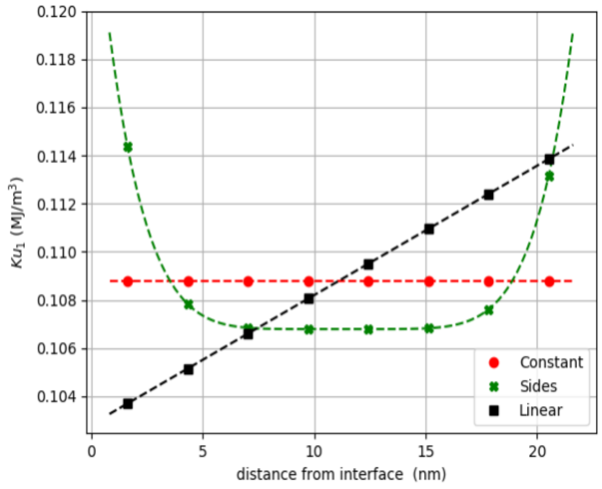

Figure S40: Vertical variation of the uniaxial anisotropy magnitude within the $Co_{2.3}Al$ film. The red curve corresponds to the homogeneous case, while the green and black curves represent two scenarios with inhomogeneously distributed anisotropy energy used in the theoretical modeling. These are labeled as "constant," "sides," and "linear," respectively.

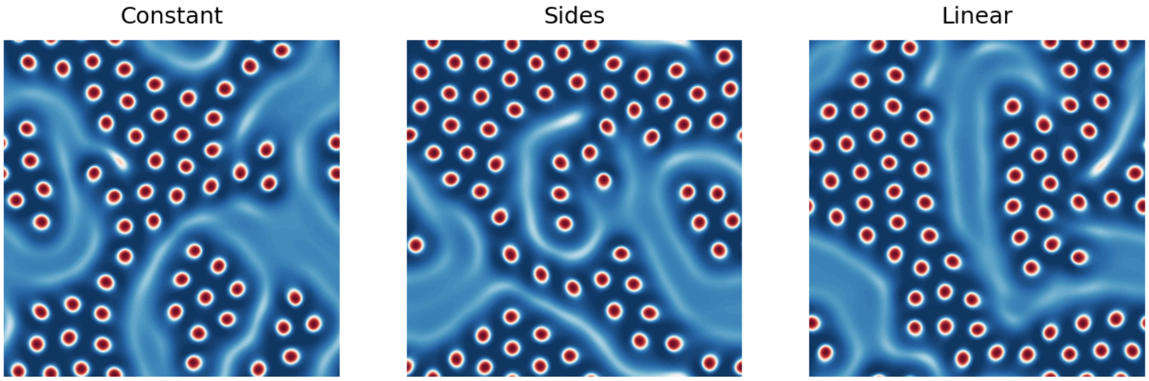

Figure S41: Normalized magnetization at $\mu_0 H_z$ = 0.3 T under the assumption of the different anisotropy distributions shown in Figure S40. The color code represents the magnetization component: Red (down), blue (up), across a cut parallel to the film surface. The size of the images is 1000×1000 nm^2.

The simulations clearly show that even the strong modification of the uniaxial anisotropy profiles within the film (Figure S40) does not have any significant influence on the simulation results, as can be seen in Figure S41. Although the calculated patterns showing the stability of the skyrmions changes in detail with regard to the distribution of the nearly in-plane magnetization (bright blue and white color), the general nature of the magnetic textures remains unchanged. Even the size of the skyrmions remains almost unchanged. In conclusion, it is a legitimate approximation to model the anisotropy as constant throughout the $Co_{2.3}Al$ layer.

References

1. Hendriksen, P. V., Linderoth, S. & Lindgård, P.-A. Finite-size modifications of the magnetic properties of clusters. *Phys. Rev. B* **48**, 7259 (1993).
2. Lee, K.-M., Choi, J. W., Sok, J. & Min, B.-C. Temperature dependence of the interfacial magnetic anisotropy in W/CoFeB/MgO. *AIP Adv.* **7** 065107 (2017).
3. Yu, X. Imaging Magnetic Vortices Including Skyrmions by Lorentz TEM and Differential Phase-Contrast Microscopy. *Microsc. Microanal.* **25**, 28-29 (2019).
4. Smits, F. M. Measurement of sheet resistivities with the four-point probe. *Bell Syst. Tech. J.* **37**, 711-718 (1958).
5. Nagaosa, N. & Tokura, Y. Topological properties and dynamics of magnetic skyrmions. *Nat. Nanotechnol.* **8**, 899-911 (2013).
6. Releases, (grahamrow/Muview2, GitHub (2023; https://github.com/grahamrow/Muview2/releases).
7. T. A. Caswell et al., *matplotlib/matplotlib: REL: v3.7.0, Feb. 13, 2023,* (2023; https://zenodo.org/record/7637593).
